# Ferroptosis-related molecular patterns reveal immune escape, inflammatory development and lipid metabolism characteristics of the tumor microenvironment in acute myeloid leukemia

**DOI:** 10.3389/fonc.2022.888570

**Published:** 2022-11-28

**Authors:** Fang-Min Zhong, Fang-Yi Yao, Jing Liu, Hai-Bin Zhang, Jing Zhang, Nan Zhang, Jin Lin, Shu-Qi Li, Mei-Yong Li, Jun-Yao Jiang, Ying Cheng, Shuai Xu, Wen Wen, Yu-Lin Yang, Xue-Ru Zhang, Xue-Xin Cheng, Bo Huang, Xiao-Zhong Wang

**Affiliations:** ^1^ Jiangxi Province Key Laboratory of Laboratory Medicine, Department of Clinical Laboratory, The Second Affiliated Hospital of Nanchang University, Nanchang, Jiangxi, China; ^2^ School of Public Health, Nanchang University, Nanchang, Jiangxi, China

**Keywords:** ferroptosis, tumor microenvironment, immune escape, inflammatory development, lipid metabolism, acute myeloid leukemia

## Abstract

**Background:**

An increasing number of studies have revealed the influencing factors of ferroptosis. The influence of immune cell infiltration, inflammation development and lipid metabolism in the tumor microenvironment (TME) on the ferroptosis of tumor cells requires further research and discussion.

**Methods:**

We explored the relationship between ferroptosis-related genes and acute myeloid leukemia (AML) from the perspective of large sample analysis and multiomics, used multiple groups to identify and verify ferroptosis-related molecular patterns, and analyzed the sensitivity to ferroptosis and the state of immune escape between different molecular pattern groups. The single-sample gene set enrichment analysis (ssGSEA) algorithm was used to quantify the phenotypes of ferroptosis-related molecular patterns in individual patients. HL-60 and THP-1 cells were treated with ferroptosis inducer RSL3 to verify the therapeutic value of targeted inhibition of *GPX4*.

**Results:**

Three ferroptosis-related molecular patterns and progressively worsening phenotypes including immune activation, immune exclusion and immunosuppression were found with the two different sequencing approaches. The FSscore we constructed can quantify the development of ferroptosis-related phenotypes in individual patients. The higher the FSscore is, the worse the patient’s prognosis. The FSscore is also highly positively correlated with pathological conditions such as inflammation development, immune escape, lipid metabolism, immunotherapy resistance, and chemotherapy resistance and is negatively correlated with tumor mutation burden. Moreover, RSL3 can induce ferroptosis of AML cells by reducing the protein level of *GPX4*.

**Conclusions:**

This study revealed the characteristics of immunity, inflammation, and lipid metabolism in the TME of different AML patients and differences in the sensitivity of tumor cells to ferroptosis. The FSscore can be used as a biomarker to provide a reference for the clinical evaluation of the pathological characteristics of AML patients and the design of personalized treatment plans. And *GPX4* is a potential target for AML treatment.

## Graphical Abstract

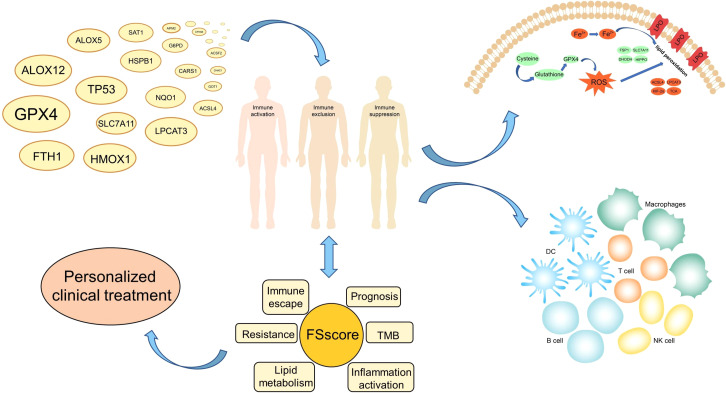

## Introduction

Cell death is important for maintaining the normal growth and development of living organisms and for maintaining homeostasis. The classic cell death modes include apoptosis, autophagy and necrosis (1). In 2012, Dixon et al. proposed a new type of iron-dependent programmed cell death induced by erastin and RSL3 and other small molecules, called ferroptosis, which is mainly characterized by the generation of reactive oxygen species (ROS), lipid peroxidation and iron accumulation (2). When a specific small molecule compound interacts with a specific target in the cell, it will cause the reduction in antioxidants such as glutathione (GSH) and glutathione peroxidase 4 (*GPX4*), and the antioxidant capacity of the cell will be weakened. In addition to a large amount of ROS accumulation, under the synergistic effect of iron, lipid peroxidation of the cell membrane induces ferroptosis (3, 4). Ferroptosis is involved in many inflammatory and immune diseases and cancers (5–7). It has potential clinical value to treat diseases by using regulators that affect the occurrence of ferroptosis. Acute myeloid leukemia (AML) is the most common hematological malignant tumor. The pathogenesis mechanism is still unclear. An increasing number of studies have revealed the relationship between ferroptosis and AML. For example, ferroptosis plays an important role in the differentiation of AML induced by ATPR (8), dihydroartemisinin (DHA) induces ferroptosis of AML cells through autophagy-dependent degradation of ferritin (9), and erastin increases the sensitivity of AML cells to chemotherapy by inducing ferroptosis (10). These studies suggest that the induction of ferroptosis has potential therapeutic value for AML.

The sensitivity of cells to ferroptosis is closely related to lipid metabolism, which induces ferroptosis through lethal lipid peroxides (LPOs) accumulated by lipid peroxidation (11). There are many types of lipids, and their biological functions are quite different. The synthesis and metabolism of lipids are precisely regulated. However, in the process of ferroptosis, polyunsaturated fatty acids (PUFAs), such as linoleic acid (LA), linolenic acid (LNA) and arachidonic acid (AA), play an important role (12); AA is particularly important, as it is very prone to peroxidation and can be oxidized to LPO (11, 13). GSH, ferroptosis suppressor protein 1 (FSP1), cystine/glutamate antiporter (system XC^-^), and *GPX4* play an important regulatory role in the mechanisms that protect cells from ferroptosis caused by oxidative stress (14–17). These characteristics all indicate that the occurrence of ferroptosis is related to complex metabolic regulation, and the destruction of cellular redox homeostasis is one of the most critical factors.

How ferroptosis is regulated by immune cells and the relationship between ferroptosis induction therapy and antitumor immunity are worthy of in-depth research and discussion. The immune system includes innate immunity and adaptive immunity (18). The effect of ferroptosis on the immune system is mainly reflected in the impact on the number and function of immune cells, as well as the specific reaction and inflammatory reaction produced by immune cells after the occurrence of ferroptosis (19). In addition to tumor cells, the tumor microenvironment (TME) is also rich in a large number of immune cells, including T cells, B cells, monocytes, macrophages, dendritic cells (DCs), natural killer (NK) cells, neutrophils and myeloid-derived suppressor cells (MDSCs) (20). Many studies have revealed the relationship between ferroptosis and immune cells. For example, *TLR2* on macrophages eliminates ferroptotic cells by recognizing phosphatidylethanolamines on ferroptotic cells (21), neutrophils maintain the inflammatory response that occurs after ferroptosis in related tissues (22), and CD8+ T cells kill cancer cells by stimulating ferroptosis (23), indicating that ferroptosis plays an important role in antitumor immunity.

Ferroptosis is more likely than apoptosis to trigger an inflammatory response. For example, ferroptotic cells express more phosphatidylserine on the plasma membrane to release “eat me” signals to induce macrophage aggregation (24). The occurrence and development of inflammation is a double-edged sword in antitumor immunity (25). Acute inflammation is conducive to the protective immunity activated in anticancer therapy, while chronic inflammation provides a favorable environment for tumor cell proliferation and immune escape. Studies have shown that cancer cells evade the immune system by producing a large number of cytokines and chemokines that inhibit immune cells. For example, in patients with melanoma, pancreatic cancer, and colorectal cancer (26–28), the expression levels of the immunosuppressive cytokines *IL-10* and *TGF-β* are significantly higher than the expression levels of the immunostimulatory cytokines *IL-2*, *IL-12* and *IFN-γ*. The predominant expression of immunosuppressive cytokines causes the TME to adopt an immunosuppressive state, which in turn helps cancer cells escape immunity (29). In the blood system, the activation of inflammatory signals in hematopoietic cells and the hematopoietic niche can significantly change the connection between hematopoietic cells and their microenvironment (30). The high expression of TNF-α, *IL-6*, *TGF-β*, *IL-8* and other proinflammatory cytokines in the bone marrow can lead to negative bone marrow hematopoietic function (31, 32). A study showed that inflammation-related cytokines inhibit the proliferation of normal progenitor cells, significantly promote the growth and survival of AML cells and are not affected by the mutation status of AML cells. The abnormal expression of cytokines creates a favorable TME for AML (33).

In summary, the occurrence of ferroptosis is closely related to biological behaviors such as the immune response, inflammation development, and lipid metabolism. However, there are few research reports on the relationship between ferroptosis and the occurrence and development of AML. As a blood tumor, AML is complicated by bone marrow cell proliferation, and changes in immune and inflammatory responses occur in the peripheral blood microenvironment at all times. Therefore, a comprehensive understanding of the TME characteristics related to ferroptosis will help us improve our understanding of the abnormal hematopoietic microenvironment of AML and provide insights for clinical diagnosis, treatment and prognostic evaluation. In this study, we integrated the genome information of 992 AML specimens, including expression profile chip data and high-throughput sequencing data, and comprehensively analyzed the characteristics of immune cell infiltration, lipid metabolism, and inflammation development in the TME of AML patients. We divided patient transcriptome data into a gene chip group (GEO group) and a high-throughput sequencing group (TCGA group). Both sets of data revealed three different ferroptosis-related phenotypes in AML patients and deeply reflected that immune escape and inflammation development in AML patients gradually worsened. To better evaluate the development of ferroptosis-related phenotypes in individual patients, we constructed a gene signature (FSscore) to quantify these phenotypes. The FSscore not only reflects the TME status of AML patients but also accurately evaluates the pathological characteristics of AML patients, such as prognosis, immunotherapy, and drug resistance. Finally, we experimentally confirmed that GPX4 is a potential target for AML treatment.

## Methods

### Data acquisition and preprocessing

The workflow of this project was shown in **Figure S1**. This study included 992 AML samples containing clinical survival information and 337 healthy control samples, including samples from six GEO (Gene-Expression Omnibus) cohorts (GSE10358, GSE12417-GPL96, GSE12417-GPL570, GSE37642, GSE71014, GSE146173), The Cancer Genome Atlas-Acute Myeloid Leukemia (TCGA-LAML) cohort, and the Genome Tissue Expression (GTEx)-whole blood cohort. For the GEO cohorts of the affymetrix platform was used, after downloading the original “CEL” file of the microarray data, we used the robust multiarray averaging (RMA) method with the “affy” package for standardization. For the microarray data of other platforms, we downloaded the normalized matrix file. For high-throughput sequencing data, we transformed the raw data into transcripts per kilobase million (TPM) values. Then, we used the combat algorithm with the “sva” package to perform batch correction on all microarray data. The normalized RNA-seq data (RSEM tpm) of the TCGA-LAML and GTEx whole blood datasets were downloaded from the UCSC XENA database (https://xenabrowser.net/datapages/). Somatic mutation data and gene copy number data were downloaded from the TCGA database (https://portal.gdc.cancer.gov/), Tumor mutation burden (TMB) calculation method: TMB = (total count of variants)/(the whole length of exons). All data were analyzed using R x64 4.1.0 and related R Bioconductor packages, and the data information is shown in **Table S1**. Sixty ferroptosis-related genes (FRGs) were retrieved from previous literature records (34) and are summarized in **Table S2**.

### Unsupervised clustering for FRGs

We used the consensus clustering algorithm *via* the “ConsensusClusterPlus” package to perform unsupervised cluster analysis on the mRNA expression of 60 FRGs (35) and performed 1000 repetitions to ensure the stability of classification, which was also verified by t-distributed stochastic neighbor embedding (t-SNE).

### Pathway enrichment analysis, functional annotation and protein–protein interaction (PPI) network analysis

Gene set variation analysis (GSVA) can quantify the activity of biological processes and signal pathways in different samples based on the expression of genes in the data set (36). We performed GSVA analysis on the “c2.cp.kegg.v2.2.symbols” gene set downloaded from the MSigDB database (37). An adjusted P value < 0.05 was regarded as statistically significant to analyze the biological behavior differences in ferroptosis-related molecular patterns. For ferroptosis-related genes and ferroptosis-related phenotype genes, we used the “clusterProfiler” package for functional annotation and uploaded them to the STRING database (https://string-db.org) to obtain their PPI network.

### Evaluation of TME immune cell infiltration level

The CIBERSORT algorithm is based on the support vector regression method to infer the proportions of various immune cells from the mixed cells of the tumor sample (38). We used an algorithm based on LM22 gene signatures to evaluate the infiltration level of immune cells such as B cells, T cells, NK cells and macrophages.

### Identification of ferroptosis-related phenotype genes

To better identify ferroptosis-related phenotypes, we adopted the empirical Bayesian approach through the “LIMMA” package to analyze the difference in gene expression between different ferroptosis-related molecular patterns (39). An adjusted P value <0.05 was used as the significance standard to determine differentially expressed genes (DEGs). The genes after the intersection of DEGs of different FRG cluster subtypes were defined as ferroptosis-related phenotype genes.

### Dimension reduction and construction of ferroptosis-related phenotype gene signatures

We tried to quantify ferroptosis-related phenotypes to better assess the degree of tumor development in AML patients. First, the ferroptosis-related phenotype genes identified by the GEO group and TCGA group were intersected, and a total of 317 overlapping genes were screened. We considered these genes to be ferroptosis-related phenotype signature genes. The phenotypic genes related to the prognosis of AML patients in the GEO group and TCGA group were further screened for dimension reduction and defined as the ferroptosis-related phenotype gene set. Single-sample gene set enrichment analysis (ssGSEA) can calculate the ferroptosis-related phenotype gene set enrichment score of individual samples, indicating the degree to which these genes are synergistically upregulated or downregulated in the sample, and the degree of development of ferroptosis-related phenotypes is positively correlated with the overall expression levels of these genes. Therefore, we used ferroptosis-related phenotype gene set enrichment scores, collectively named the FSscore, to quantify ferroptosis-related phenotypes.

### Correlation analysis of ferroptosis-related molecular patterns and other biological characteristics, such as immune escape, lipid metabolism, and inflammation development

To explore the sensitivity of different molecular patterns to the occurrence of ferroptosis, we collected lipid metabolism-related signaling pathways and other gene sets that have been confirmed to be related to ferroptosis through the MSigDB database. To determine the degree of immune escape and inflammation development of molecular patterns with different phenotypes, we used a series of gene sets designed by Mariathasan et al. (40), including immune checkpoint, angiogenesis, nucleotide excision repair, DNA damage repair, mismatch repair gene sets and gene sets related to cell adhesion, tumor angiogenesis, and inflammatory response signaling pathways from the MSigDB database. MDSCs are closely related to tumor immune escape (41). From the study of Charoentong et al., we obtained the marker genes of MDSCs (42). Finally, we used the tumor immune dysfunction and exclusion (TIDE) website (http://tide.dfci.harvard.edu/) to predict the TIDE score of samples with different ferroptosis-related phenotypes to verify the immune escape level (43).

### Immune checkpoint blockade response, drug sensitivity prediction, and small molecule drug screening

We collected the genomic and clinical information of two immunotherapy groups: an anti-PD-L1-treated advanced urothelial cancer cohort (IMvigor210) (40) and an anti-PD-1-treated metastatic melanoma cohort (GSE78220) (44). The gene expression data of the two cohorts were transformed to TPM values. The Genomics of Drug Sensitivity in Cancer (GDSC; https://www.cancerrxgene.org/) database was used to predict the sensitivity of all patients to 138 chemotherapy drugs, and the “pRRophetic” package was used to calculate the value of half-maximal inhibitory concentration (IC50) (45, 46). Then, we uploaded the genes that were upregulated and downregulated in immunosuppression phenotypes to the CMap database (47) and used the mode-of-action (MoA) analysis function of the website to predict the potential small molecule drugs to regulate ferroptosis-related phenotypes and targets to induce therapeutic effects.

### *In vitro* assays

Human AML cell lines HL-60 and THP-1 were cultured in RPMI1640 medium containing 10% fetal bovine serum and 1% penicillin-streptomycin at 37°C and 5% CO2. Cell counting Kit (CCK-8) (Bioss, BA00208, USA) was used to evaluate cell viability. The cells were inoculated into 96 well flat bottom microtiter plates with a density of 20000 cells per well, and then treated with different concentrations of RSL3 or (and) ferrostatin-1 for 48 hours. After that, 10 μl CCK-8 reagent was added to each well and incubated at 37 °C for 2.5 h. The absorbance of the cells at 450 nm was measured by microplate reader. Western blot analysis was used to detect the expression of GPX4. Antibodies used were rabbit anti-GAPDH (1: 1000, #5174) from Cell Signaling Technology (Danvers, MA, USA) and anti-GPX4 (1:1000, T56959) from Abmart (Shanghai, China).

### Statistical analysis

The Wilcoxon rank-sum test and Kruskal–Wallis test were used to determine the difference between two groups and multiple groups, respectively. The “survminer” package was used to determine the cutoff point of various scores and divide patients into high and low groups. The log rank test was used to determine the significance of Kaplan–Meier survival analysis. Univariate Cox regression analysis was used to calculate the hazard ratios (HRs). Multivariate Cox regression analyses further determined independent prognostic factors. The “forestplot” package was used for univariate and multivariate independent prognostic analysis. The specificity and sensitivity of the FSscore were evaluated by receiver operating characteristic (ROC) curve analysis, and the “pROC” package was further used to determine the area under the curve (AUC). The “maftools” package was used to show the characteristics of somatic mutations in TCGA-LAML patients. The chromosomal location where the copy number variation in FRGs occurred was described with the “RCircos” package. A two-sided p value < 0.05 was considered to indicate statistical significance.

## Results

### Variation landscape of FRGs in AML

**Figure 1:**
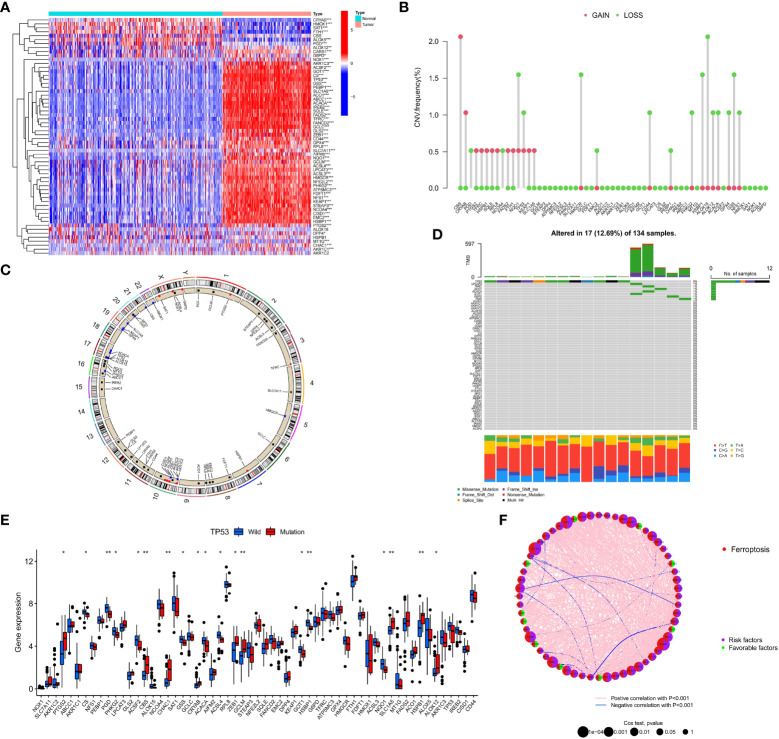
Genetic characteristics of FRGs in AML patients. **(A)** The heatmap depicts the difference in the expression of 60 FRGs in AML samples and normal samples. Wilcoxon test, *P < 0.05; **P < 0.01; *P < 0.001. **(B)** CNV frequency of FRGs in the TCGA cohort. **(C)** The position of FRGs on 23 chromosomes where CNV occurred in the TCGA cohort. **(D)** Somatic mutations of 60 FRGs in 134 TCGA-LAML patients. Each column in the waterfall diagram represents the mutation type of each patient, the upper part shows the TMB of each patient, and the right side shows the mutation frequency and mutation type ratio of FRGs. The ratio of different base transitions is shown below. **(E)** The expression of 60 FRGs in *TP53* mutation-type patients and wild-type patients, Wilcoxon test, *P < 0.05; **P < 0.01. **(F)** The interaction of FRGs in AML patients and its relationship with prognosis. Spearman correlation analysis was used to calculate the correlation between FRGs; p<0.001 indicates correlation, pink lines represent a positive correlation, and blue lines represent a negative correlation. Univariate Cox regression analysis was used to calculate the HRs to identify the relationship between FRG expression and prognosis. HR<1: Favorable factors for prognosis, indicated by a green semicircle. HR>1: Risk factors for prognosis, indicated by a purple semicircle. The size of the circle indicates the degree of association between the gene and the prognosis. The larger the circle is, the stronger the association with the prognosis.

The phenotype of organisms is mainly regulated by gene expression, and the occurrence of ferroptosis is no exception. To explore the relationship between ferroptosis and AML, we first analyzed the genetic characteristics of FRGs in AML cells. Based on the transcriptome sequencing data of tumor samples and normal blood samples from AML patients, we observed that most FRGs were upregulated in AML (**Figure 1A**). The high expression of these genes may play an important role in the occurrence and development of AML. We further analyzed the copy number variation (CNV) frequency of FRGs in AML patients, and 27 genes had copy number gain or loss. High expression of *PTGS2, TFRC, HSPB1, SQLE, RPL8, NCOA4, FADS2, KEAP1*, and *SLC1A5* may be related to an increase in CNV frequency (**Figure 1B**). **Figure 1C** shows the location of FRGs on the chromosome where CNVs occurred. The occurrence of leukemia is closely related to gene mutations, and we summarized the somatic mutations of FRGs. Among 134 samples, 17 had gene mutations, and TP53 had the highest mutation frequency (**Figure 1D**). To better explore the interaction between these genes, we further analyzed the difference in the expression of FRGs between *TP53* mutation-type and wild-type patients, the correlations among FRG expression levels, and the prognostic value of FRGs in AML patients. The results showed that compared with that in the *TP53* wild-type patient group, the expression of 9 FRGs, such as *PTGS2*, was upregulated in the mutant patient group, and the expression of 10 FRGs, such as *PGD*, was downregulated (**Figure 1E**). Correlation analysis showed that the expression levels of most FRGs were positively correlated; for example, *TP53* expression was positively correlated with the expression of other FRGs (**Figure 1F** and **Table S3**). Univariate Cox regression analysis showed that 10 FRGs such as *TP53* and *PHKG2*, were favorable factors in terms of the prognosis of AML (**Figure 1F** and **Table S4**). High expression of these genes indicated a better prognosis for AML patients. The remaining FRGs were risk factors. The mutation of *TP53* in AML often indicates a poor prognosis, poor cytogenetic risk and immunosuppression (48, 49). In connection with the differential expression of FRGs in AML and normal samples, we observed that *PTGS2, CBS, CHAC1, GCLM, SLC1A5, HSPB1*, and *ALOX12* were highly expressed in AML samples and patients with *TP53* mutations and also showed significant positive correlations in terms of expression levels, indicating that they are also risk factors in terms of the prognosis of AML patients.

Based on the above results, we observed that the genetic changes in FRGs in AML samples versus normal samples, including gene structure, number, and expression, were highly heterogeneous, indicating that FRGs may have a profound impact on the occurrence and development of AML.

### Identification of ferroptosis-related molecular patterns and analysis of their biological characteristics

**Figure 2:**
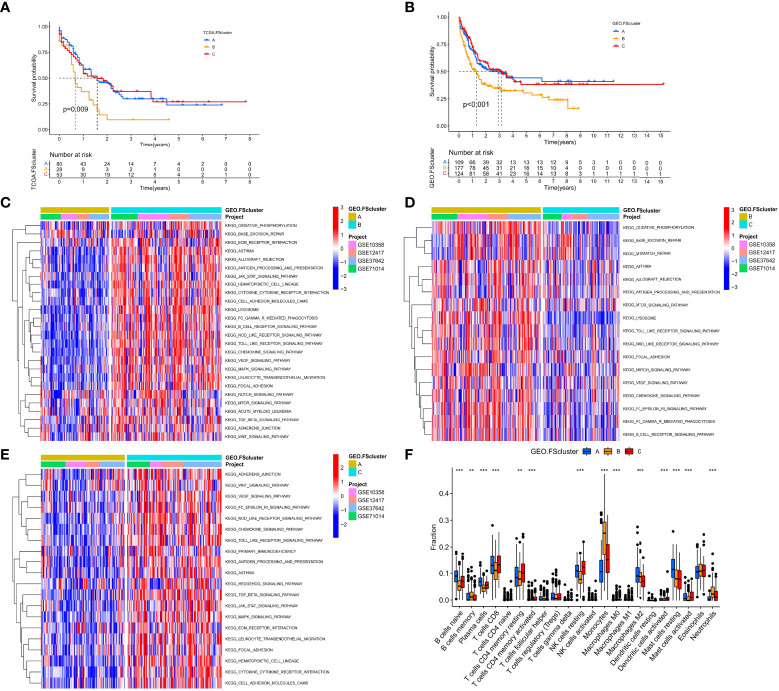
Survival analysis and biological characteristics analysis of ferroptosis-related molecular patterns. **(A)** Kaplan–Meier survival analysis of different ferroptosis-related molecular pattern groups in the TCGA cohort, log rank test. **(B)** Kaplan–Meier survival analysis of different ferroptosis-related molecular pattern groups in the GEO cohort, log rank test. **(C–E)** GSVA showed the activation levels of biological pathways in different ferroptosis-related molecular pattern groups in the GEO cohort. **(F)** The infiltration ratio of various TME cells in three ferroptosis-related molecular pattern groups in the GEO cohort, Kruskal–Wallis test, **P < 0.01; ***P < 0.001.

To further analyze the influence of FRGs on the biological functions of AML, we first performed Kyoto Encyclopedia of Genes and Genomes (KEGG) enrichment analysis and found that these genes are involved in many signaling pathways related to lipid metabolism, energy metabolism and ferroptosis (**Figure S2A**). The PPI network also shows that FRGs have complex interactions at the protein level (**Figure S2B**). The high correlation between FRGs at the mRNA and protein expression levels indicates that the combined effect of these genes may have an important impact on the biological process of AML. Therefore, we performed unsupervised clustering based on the expression of FRGs in the TCGA cohort. The clustering results showed that AML patients in the TCGA cohort were divided into three different molecular patterns, named TCGA.FScluster A-C (**Figure S2C**, and **Table S5**), and t-SNE verified the stability of the clustering results (**Figure S2D**). We observed that most of the FRGs in FScluster B were upregulated (**Figure S2E**). Survival analysis showed that TCGA.FSclusters A and C were related to a better prognosis, while TCGA.FScluster B was related to a worse prognosis (**Figure 2A**). To verify these clustering characteristics, we expanded the number of patients and performed the same analysis on the microarray data of AML patients in the GEO database. Since GSE146173 contains high-throughput sequencing data, expression data for some of the FRGs is missing in GSE37642-GPL96. These two sets of data were used for verification. We merged only the remaining AML chip data (GSE12417-GPL570, GSE10358, GSE37642, GSE71014) for the GEO group. We clustered the GEO group and obtained three completely different subgroups, named GEO.FScluster A-C (**Figures S2F, G** and **Table S6**) and verified by t-SNE (**Figure S2H**). Survival analysis showed that patients in GEO.FScluster B had a worse prognosis, while patients in GEO.FSclusters A and C had a better prognosis (**Figure 2B**).

We further analyzed the biological characteristics of the molecular patterns. GSVA showed that compared with FScluster A, FSclusters B and C were enriched a large number of inflammatory immune and cancer-promoting signaling pathways in both the TCGA group and the GEO group, and the enrichment degree of FScluster B was higher than that of FScluster C; the enriched pathways included the chemokine signaling pathway, cytokine–cytokine receptor interaction, the NOD-like receptor signaling pathway, the Toll-like receptor signaling pathway, the TGFβ signaling pathway, the JAK-STAT signaling pathway, cell adhesion and the MAPK signaling pathway (GEO group: **Figures 2C–E** and **Table S7**, TCGA group: **Figures S3A–C** and **Table S8**). In the past, inflammatory immune pathways were thought to be limited to immune cells activating immune responses, but an increasing number of studies have shown that TLRs, NLRs, chemokines and cytokines highly expressed by tumor cells can promote immune escape (32, 50, 51). Further analysis of TME cell infiltration showed that FSclusters A and C in the GEO group and TCGA group showed a large amount of innate immune cell infiltration, including NK cells, eosinophils, mast cells, dendritic cells and adaptive immune cells. Infiltrations included naïve B cells, CD8+ T cells, and resting memory CD4+ T cells (GEO group: **Figure 2F** and **Table S9**, TCGA group: **Figure S3D** and **Table S10**). FScluster B showed increased infiltration of memory B cells, monocytes, M2 macrophages and neutrophils. Both FScluster A and C patients with immune activation showed a better prognosis, while the high infiltration of inflammatory cells in FScluster B indicated the deterioration of the tumor’s inflammatory microenvironment and the suppression of the patient’s immune function, which are indicators of a poor prognosis.

### Analysis of the sensitivity of different molecular patterns to the occurrence of ferroptosis

**Figure 3:**
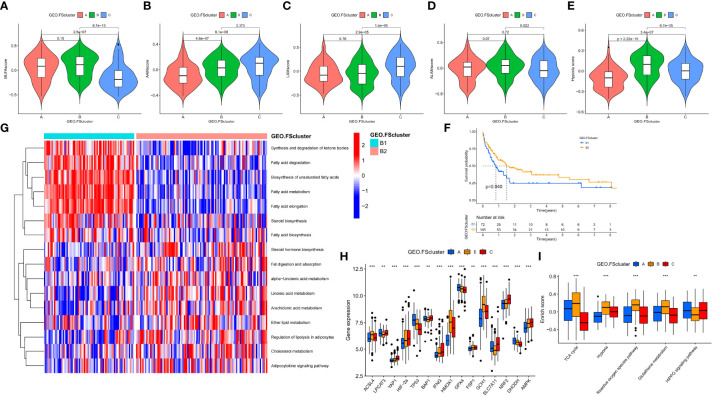
Analysis of the metabolic characteristics of unsaturated fatty acids in ferroptosis-related molecular patterns and other factors affecting ferroptosis. **(A–E)** Differences in BUFAscore, AAMscore, LAMscore, ALAMscore, and hypoxia score in different ferroptosis-related molecular pattern groups in the GEO cohort, Kruskal–Wallis test. **(F)** Kaplan–Meier survival analysis of different cluster subtypes in the FScluster B of the GEO group, log rank test. **(G)** GSVA showed the activation levels of lipid metabolism pathways in different cluster subtypes. FScluster B of the GEO group. **(H, I)** Differences in genes and signaling pathways that affect ferroptosis among different ferroptosis-related molecular patterns, Kruskal–Wallis test, **P < 0.01; ***P < 0.001.

To explore the relationship between tumor cells and ferroptosis in AML patients with different TMEs and prognostic status, we further analyzed various factors that affect the occurrence of ferroptosis. The occurrence of ferroptosis is often accompanied by abnormal lipid metabolism. We examined the characteristics of lipid metabolism among different ferroptosis-related molecular pattern groups. In the GEO and TCGA cohorts, we observed that FScluster B had the strongest lipid metabolism, while FScluster A had the weakest lipid metabolism (GEO group: **Figures S4A–C** and **Table S11**, TCGA group: **Figures S5A–C** and **Table S12**). We conducted in-depth analysis on the GEO group with a larger number of patients and found that the fatty acid metabolism, degradation, elongation signaling and unsaturated fatty acid biosynthesis signaling pathways were significantly activated in FSclusters A and B compared to FScluster C. The occurrence of ferroptosis depends on phospholipids containing polyunsaturated fatty acid chains (PUFA-PL). The biosynthesis of unsaturated fatty acids creates conditions for the occurrence of ferroptosis. Polyunsaturated fatty acids mainly include linoleic acid, linolenic acid and arachidonic acid. We also observed that the metabolism of linoleic acid and arachidonic acid was enhanced in FScluster C. Based on the results of GSVA, we further constructed four lipid metabolism scores for unsaturated fatty acid biosynthesis, linoleic acid metabolism, α-linolenic acid metabolism and arachidonic acid metabolism, and named them the BUFAscore, LAMscore, ALAMscore and AAMscore, respectively (**Table S13**). Kruskal–Wallis test results showed that BUFAscore was higher in FSclusters A and B, the AAMscore and LAMscore were higher in FScluster C, and the ALAMscore was only slightly upregulated in FScluster B (**Figures 3A–D**). These lipid metabolism characteristics indicate that FSclusters A and B may be more sensitive to the occurrence of ferroptosis. Taken together, the biological characteristics of each subtype indicate that the massive infiltration of immune cells in FScluster A may induce ferroptosis. Although FScluster B was represented by immunosuppression, the worsening of inflammation confers a hypoxic and ROS-enriched TME, which in turn promotes lipid peroxidation and increases the sensitivity of tumor cells containing high levels of unsaturated fatty acids to ferroptosis. We constructed a hypoxia score based on the enrichment analysis of the hypoxia signaling pathway (**Table S13**). The difference analysis showed that the hypoxia score was the highest in FScluster B (**Figure 3E**). This result verified our conjecture. In the above, we observed the activation of the tumor cell inflammatory immune pathway in FScluster C, which may promote the immune escape of tumor cells, prevent the occurrence of ferroptosis induced by immune cells, and a low degree of hypoxia in the weak inflammatory environment of FScluster C, so the tumor cells in FScluster C are more resistant to ferroptosis.

We also analyzed the relationship between BUFAscore and patient prognosis in each ferroptosis-related molecular pattern group. Patients with a high BUFAscore in FSclusters A and B had poor survival (**Figures S6A, B**). In FScluster C, patients with a high BUFAscore had better survival, but there was no significant difference (**Figure S6C**). Patients in FScluster B have poor prognostic characteristics, and we analyzed the relationship between lipid metabolism and prognosis in this molecular pattern. By unsupervised clustering of lipid metabolism signaling pathway enrichment scores, we further divided FScluster B into FSclusters B1 and B2 (**Figures S6D, E**). Survival analysis showed that patients in FScluster B1 had a worse prognosis (**Figure 3F**). Lipid metabolism pathway enrichment analysis showed that fatty acid metabolism and synthesis-related signaling pathways and unsaturated fatty acid biosynthesis signaling pathways were activated in FScluster B1, and unsaturated fatty acid metabolism signaling pathways, including linoleic acid, α-linolenic acid and arachidonic acid pathways, were significantly enriched in FScluster B (**Figure 3G**). These results indicate that with the deterioration of the TME in patients with AML, the tumor cells of patients with poor prognosis exhibit enhanced fatty acid metabolism and unsaturated fatty acid biosynthesis, and the enhanced metabolism of unsaturated fatty acids suggests a better prognosis for patients.

To better assess the propensity for the occurrence of ferroptosis between different molecular patterns, we summarized the genes and signaling pathways that promote and inhibit ferroptosis through existing research (5). *ACSL4, LPCAT3, YAP1, HIF-2α, TP53, BAP1, IFNG*, and *HOMOX1* and the TCA cycle, hypoxia, reactive oxygen signaling pathway and glutathione metabolism signaling pathways have been proven to promote the occurrence of ferroptosis. *GPX4, FSP1, GCH1, SLC7A11, NRF2, DHODH, AMPK* and the HIPPO signaling pathway have inhibitory effects. The results of the Kruskal–Wallis test showed that *ACSL4*, *HMOX1*, and *GCH1* were highly expressed in FScluster B, while FScluster A mainly showed high expression of ferroptosis inhibitory genes such as *GPX4* and DHODH. FScluster C was characterized by low expression of the ferroptosis-promoting genes *YAP1*, *HIF-2α*, and *IFNG*, and high expression of the ferroptosis-suppressor genes *SLC7A11, NRF2*, and AMPK (GEO group: **Figure 3H**, TCGA group: **Figure S7A**). The ferroptosis signaling pathway was highly activated in FScluster B, and the activity of inhibiting the ferroptosis signaling pathway was significantly reduced; the opposite was true for FSclusters A and C (GEO group: **Figure 3I** and **Table S14**, TCGA group: **Figure S7B** and **Table S15**). These results indicate that the expression of FRGs in ferroptosis-related molecular patterns is specific, and the connection between these genes and the relationship between these genes and ferroptosis in AML needs further discussion and research. However, ferroptosis-related pathways show a high degree of consistency. In cluster B, which was associated with the worst prognosis, the proferroptosis pathways were uniformly activated, while the antiferroptosis pathways were inhibited. These findings combined with the characteristics of lipid metabolism indicate that tumors cells in FScluster B have a higher tendency to undergo ferroptosis than those in other clusters.

### Phenotypic analysis of ferroptosis-related molecular patterns

To better identify and verify the phenotypes of the three ferroptosis-related molecular patterns, we first identified the DEGs shared between the molecular patterns. A total of 1883 and 1222 shared DEGs were identified in the GEO and TCGA groups, respectively (**Figures S8A, B**). In the GEO group and TCGA group, three different genomic subtypes were further identified through unsupervised clustering and were named GEO.geneCluster A-C and TCGA.geneCluster A-C (**Figures S8C–F** and **Tables S5, S6**). This indicates that there are indeed three molecular patterns in AML patients. We analyzed the biological and clinical characteristics of genomic subtypes. We found that the three genomic subtypes were very similar to the previously identified ferroptosis-related molecular patterns. In the GEO group, we observed that FScluster A and geneCluster A had a high degree of overlap. Some of the samples in FScluster B and in FScluster C constituted geneCluster C, and the remaining samples in FScluster B were classified into geneCluster B (**Figure S9A**). In the TCGA group, some samples in FScluster A corresponded to geneCluster A, some samples in FScluster C and FScluster B were in geneCluster B, and the remaining FScluster A and C samples formed geneCluster C (**Figure S9B**). These results seem to suggest that AML samples are divided into three biological states based on the nodes of two biological processes. Through survival analysis, it was found that in the GEO and TCGA groups, the patients with samples in geneCluster A had a better prognosis, and the patients with samples in geneCluster B and C had a worse prognosis; the patients with samples in geneCluster C were mostly derived from FScluster C but showed a worse prognosis (**Figures S9C, D**). We performed GSVA and TME cell infiltration analysis again on genomic subtypes, and the results showed similar biological characteristics to ferroptosis-related molecular patterns (**Figures S10A–D, S11A–D**). Samples in geneClusters A and C were characterized by a large number of infiltrating immune cells, and those in geneCluster B presented an immunosuppressive state and highly activated inflammation status. Samples in geneCluster C also showed enrichment of a large number of inflammatory and immune signaling pathways. Based on this, we reasonably hypothesized that the changes in the inflammatory microenvironment in geneCluster C versus geneCluster A promote further immune escape of tumor cells and that the cells in this cluster are different from solid tumor cells that can directly pass through the surrounding matrix to block the attack of immune cells. As a hematological tumor, AML creates favorable survival conditions by reshaping the bone marrow microenvironment. These changes may be reflected in the promotion of tumor angiogenesis and abnormal adhesion to the niche.

**Figure 4:**
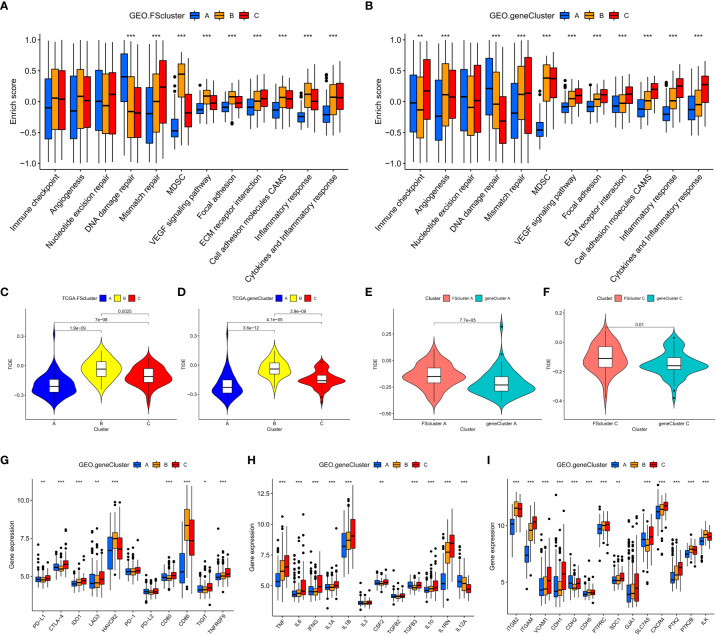
Analysis of TME characteristics associated with ferroptosis-related phenotypes. **(A, B)** Differences in pathway signatures related to tumor development and the infiltration level of MDSCs in the TME of ferroptosis-related molecular pattern and genomic subtype groups in the GEO cohort. **(C–F)** Differences in TIDE scores of ferroptosis-related molecular pattern and genomic subtype groups in the TCGA cohort. **(G–I)** Differences in gene signatures related to tumor development in the TME of ferroptosis-related molecular pattern and genomic subtype groups in the GEO cohort, G: Immune checkpoint-related genes, H: cytokines related to inflammation associated with AML, I: cell adhesion molecules associated with AML. Kruskal–Wallis test, *P < 0.05; **P < 0.01; ***P < 0.001.

Subsequent analysis showed that AML cells in geneCluster C exhibited high expression of immune checkpoints, stimulated tumor angiogenesis, highly activated cell adhesion-related and inflammation-related signals, and more MDSC infiltration than cells characterized by other ferroptosis-related molecular patterns. These malignant changes were more obvious in the genomic subtypes (GEO group: **Figures 4A, B** and **Table S16**, TCGA group: **Figures S12A, B** and **Table S17**). These results show that compared with that in cells of geneCluster A, the AML TME in cells of geneCluster C was further deteriorated, the immune escape of AML cells was enhanced, and the expression level of DEGs shared between the ferroptosis-related molecular patterns was positively correlated with the immune and inflammatory microenvironment characteristics. Further unsupervised clustering rearranged AML patient samples and divided them into three more accurate phenotypes, which we defined as the following ferroptosis-related phenotypes: The immune activation phenotype, corresponding to FScluster A and geneCluster A, was characterized by innate immune cell and adaptive immune cell infiltration and the best patient prognosis. The immune exclusion phenotype, corresponding to FScluster C and geneCluster C, was characterized by innate and adaptive immune cell infiltration and an inflammatory microenvironment, and with the development of inflammation, immune escape increased; patient prognosis for this phenotype ranked second. The immunosuppression phenotype, corresponding to FScluster B and geneCluster B, was characterized by a high degree of inflammatory cell infiltration and inflammatory microenvironment development, with the strongest immune escape ability, and corresponded to the worst prognosis in patients. To verify the characteristics of immune escape of the three ferroptosis-related phenotypes, we analyzed a TCGA dataset, which included RNA-seq data, on the TIDE website. Through the differential analysis of the calculated TIDE scores, we found that among the ferroptosis-related molecular patterns and the genomic subtypes, cluster B had the highest score, cluster C scored second, and cluster A scored the lowest (**Figures 4C, D**). This result verified the immune escape characteristics of the ferroptosis-related phenotypes. We also calculated the difference in TIDE scores between FScluster C and geneCluster C and found that the TIDE score of FScluster C was higher (**Figure 4E** and **Table S18**). This is because some samples in FScluster C and some samples in FScluster A with better ferroptosis-related phenotypes constituted geneCluster C, so the overall biological status of patients with sample in geneCluster C was better than that of patients with samples in FScluster C, while the immune escape ability was weaker. Similarly, the immune escape ability of geneCluster A was also weaker than that of FScluster A (**Figure 4F**). These results can be explained by the distribution characteristics of AML patients with different phenotypes in each cluster.

### Transcriptome characteristics of ferroptosis-related phenotypes

To further explore the relationship between ferroptosis-related phenotypes and immunity and inflammation. We analyzed the mRNA expression of related cytokines and chemokines in genomic subtypes. It has been reported in the literature that *PD-L1, CTLA-4, IDO1, LAG3, HAVCR2, PD-1, PD-L2, CD80, CD86, TIGIT* and *TNFRSF90* are considered immune checkpoint-related genes (40); *TNF, IL6, IFNG, IL1A, IL1B, IL3, CSF2, TGFB1, IL10, ILRN*, and *IL2A* are considered to be cytokines related to inflammation associated with AML (52); *TGFB2, ITGAM, VCAM1, CDH1, CDH2, CDH5, PTPRC, SDC1, GJA1, SLC7A5, CXCR4, PTK2, PTK2B*, and *ILK* are considered to be cell adhesion molecules associated with AML (53). We observed that genes related to immune checkpoints, inflammatory cytokines and cell adhesion molecules were highly expressed in geneClusters B and C (GEO group: **Figures 4G–I**, TCGA group: **Figures S12C–E**). These results once again indicate that there are indeed ferroptosis-related phenotypes with obvious immune and inflammatory characteristics in the occurrence and development of AML. Promoting inflammation development and cell adhesion-related signaling pathways and related genes are highly activated or highly expressed in tumor cells of patients with an immune exclusion phenotype, because patients with this phenotype have more innate and adaptive immune cell infiltration in the TME, and tumor cells show high activity of these signaling pathways to avoid the attack of immune cells. Although these pathways are also obviously activated in AML cells of patients with an immunosuppression phenotype, their activation degree is lower than that in AML cells of the immune exclusion phenotype. This may be because immune cells with this phenotype are suppressed and there is a high degree of inflammation to promote immune escape. Therefore, there is no need to overactivate these signaling pathways or overexpress these genes.

### Quantification of ferroptosis-related phenotypes and analysis of associated clinical characteristics

**Figure 5:**
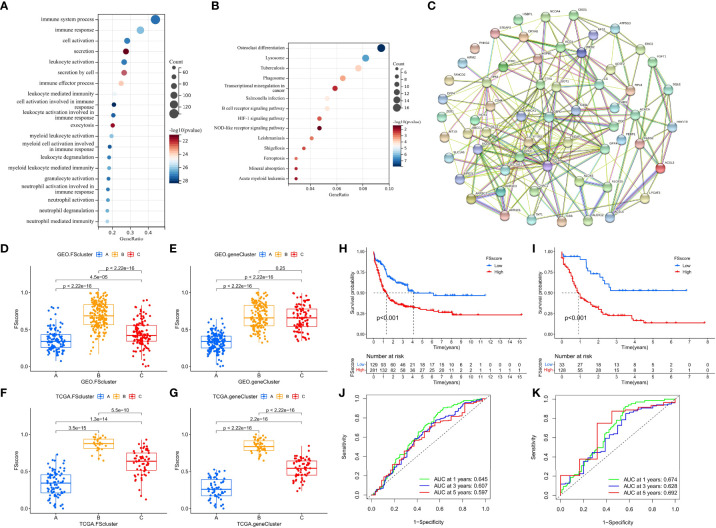
Analysis TME characteristics associated with ferroptosis-related phenotypes. **(A, B)** GO annotation and KEGG pathway enrichment analysis showed the functions of the ferroptosis-related phenotype signature genes. **(C)** PPI network of ferroptosis-related phenotype signature genes. **(D–G)** Differences in the FSscore of ferroptosis-related molecular patterns and genomic subtypes in GEO and TCGA groups, Kruskal–Wallis test. **(D–I)** Differences in the survival of patients in high and low FSscore groups in the GEO and TCGA cohorts, **(H)**: GEO group, **(I)**: TCGA group, log rank test. **(J, K)** The ROC curves showed the specificity and sensitivity of the FSscore; **(J)**: GEO group, **(K)**: TCGA group.

**Figure 6:**
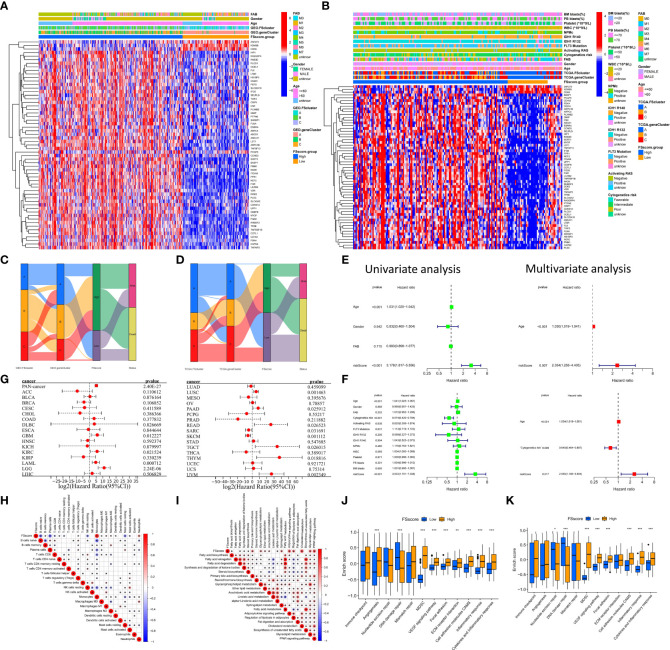
Distribution characteristics and prognostic value analysis of the FSscore, correlation analysis between characteristics of the TME and FSscore. **(A, B)** Differences in patient distribution, clinicopathological factors and phenotype signature gene expression of the high and low FSscore groups in the GEO and TCGA cohorts, the blank in the annotation of FSscore group in the TCGA cohort is due to the lack of clinical information of corresponding patients. **(C, D)** Alluvial diagram showing the changes in ferroptosis-related molecular patterns, genomic subtypes, FSscore and patient status. **(E, F)** Univariate and multivariate independent prognostic analysis of FSscore, E: GEO group, F: TCGA group. **(G)** Prognostic value of the FSscore in pancancer datasets. **(H)** Correlation analysis between the levels of TME cell infiltration and the FSscore in the GEO group. **(I)** Correlation analysis between levels of lipid metabolism and the FSscore in the GEO group. **(J, K)** Differences in pathway signatures related to tumor development and the infiltration level of MDSCs in the TME in the high and low FSscore groups. D: GEO group, E: TCGA group, Wilcoxon test. *P < 0.05; **P < 0.01, ***P < 0.001.

The ferroptosis-related phenotypes of AML patients indicate a pathological state that gradually deteriorates from immune activation to immune exclusion to immune suppression. However, the phenotype can only qualitatively assess patient stage. To better evaluate the tumor development of individual patients with AML, we used the ssGSEA method to calculate the enrichment score of the ferroptosis-related phenotype gene signatures to quantify the ferroptosis-related phenotypes with a metric named the FSscore. Gene ontology (GO) and KEGG analyses showed that these genes were closely related to immunity and inflammation (**Figures 5A, B**), and the PPI network also showed a high degree of interaction (**Figure 5C**). The Kruskal–Wallis test compared the differences in FSscore between different ferroptosis-related molecular patterns and between different genomic subtypes (**Figures 5D–G**). Patients with an immune activation phenotype had the lowest FSscore, followed by patients with an immune exclusion phenotype, and patients with an immunosuppression phenotype had the highest FSscore. Kaplan–Meier curve survival analysis was performed for AML patients divided into high FSscore and low FSscore groups based on the cutoff value identified by the survminer package. Among the GEO and TCGA groups, the prognosis of patients in the low FSscore group was significantly better than that of patients in the high FSscore group [GEO group, HR 3.178 (1.817-5.556); TCGA group, HR 3.533 (1.701-3.338)] (**Figures 5H, I**). ROC curve analysis showed that the FSscore can accurately predict the prognosis of AML patients (**Figures 5J–K**). The heatmap showed that the low FSscore group matched FScluster A and geneCluster A, and the high FSscore group corresponded to FScluster B-C and geneCluster B-C (**Figures 6A, B**). The alluvial diagram showed the differences in the attributes of individual patients (**Figures 6C, D**). These results shows that the FSscore can quantify the ferroptosis-related phenotypes of AML patients well.

Next, to further evaluate the ability of the FSscore to predict the prognosis of patients, we conducted an independent prognostic analysis of the FSscore together with other clinical characteristics of AML patients. Both univariate and multivariate independent prognostic analyses confirmed that the FSscore is an independent and reliable prognostic marker (**Figures 6E, F**). We verified the prognostic value of the FSscore in all independent cohorts of GEO group [GSE10358, HR 2.34 (0.80-6.86); GSE12417-GPL570, HR 2.60 (0.79-8.52); GSE37642, HR 2.81 (1.17-6.77); GSE71014, HR 6.93 (1.88-25.57)] (**Figures S13A–D**). We also verified the results in the other two sets of AML transcriptome data. Both chip data (GSE12427-GPL96, HR 0.97 (0.41-2.34)) and high-throughput sequencing data (GSE146173, HR 1.50 (0.67-3.39)) showed that patients with a high FSscore had a poorer prognosis than patients with a low FSscore (**Figures S13E, F**). Finally, we evaluated the prognostic value of the FSscore in 33 TCGA pancancer datasets including 10496 tumor samples. Although the analysis results showed some differences, the prognosis of patients in 12 TCGA tumor cohorts could be accurately predicted (**Figure 6G**). These results all show that the FSscore can be used as a good prognostic marker.

We also assessed the correlations among known signatures such as immune infiltration, lipid metabolism, inflammation and FSscore. The infiltration of monocytes, M2 macrophages, resting dendritic cells, and neutrophils showed a significant positive correlation with the FSscore, while the infiltration of B cells, T cells, mast cells, NK cells, and mast cells was negatively correlated with the FSscore (GEO group: **Figure 6H**, TCGA group: **Figure S13G**). The activity of most lipid metabolism pathways was positively correlated with the FSscore (GEO group: **Figure 6I**, TCGA group: **Figure S13H**). In the high FSscore group, pathways such as immune checkpoints, tumor angiogenesis, adhesion, and inflammation were activated, and MDSCs were highly infiltrated. In the low FSscore group, the activity of DNA damage repair signaling pathways was significantly increased (**Figures 6J, K**). These results clearly show that a low FSscore is closely related to immune activation, and a high FSscore is related to inflammatory cell infiltration, lipid metabolism activation, deterioration of the TME, and the enhanced immune escape ability of tumor cells through adhesion, which suggests a harsh TME. In summary, the FSscore can well assess the development status of ferroptosis-related phenotypes in AML patients and has profound guiding significance for judging the individual characteristics and clinical treatment outcomes of AML patients.

### The relationship between clinicopathological factors, tumor somatic mutations and ferroptosis-related phenotypes in AML patients

**Figure 7:**
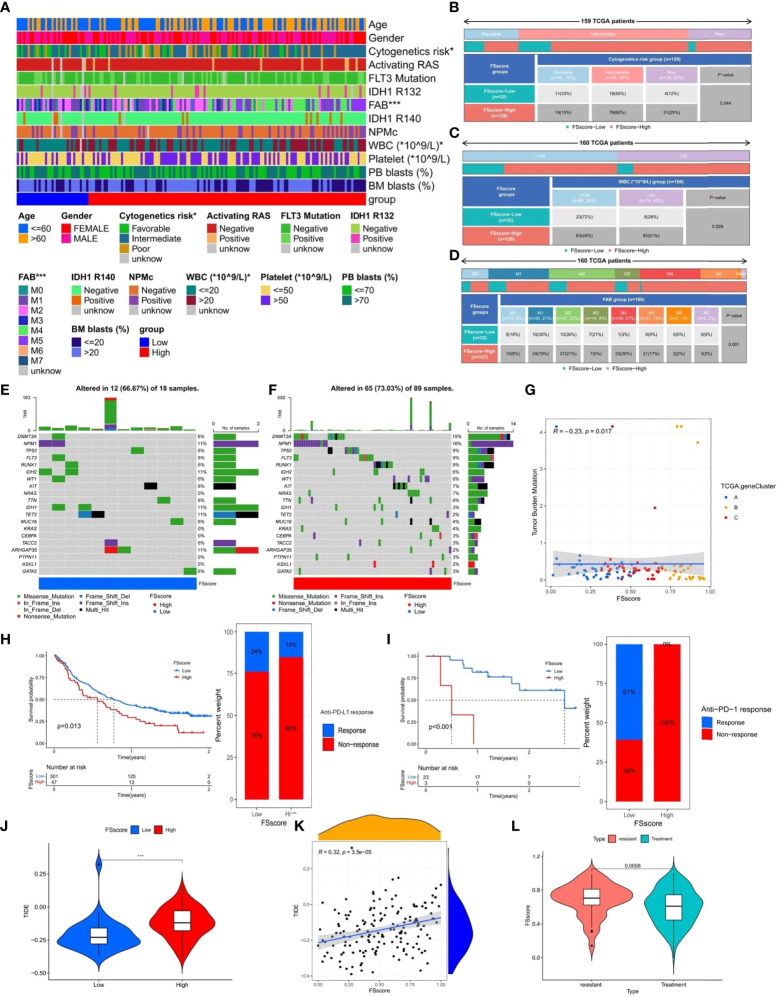
The relationship between clinicopathological factors, tumor somatic mutations, immunotherapy and FSscore. **(A–D)** Correlation analysis between clinicopathological factors and FSscore in the TCGA group, Fisher’s exact test, *P < 0.05. **(E, F)** The characteristics of tumor somatic mutations in the high and low FS score groups of the TCGA group, WBC: white blood cell, PB: peripheral blood, BM: Bone marrow, IDH: isocitrate dehydrogenase, NPMc: nucleophosmin. **(G)** Correlation analysis between TMB and FSscore in the TCGA group. **(H, I)** Differences in survival of patients and the proportion of patients in high and low FSscore groups of immune checkpoint inhibitor treatment cohorts (H: IMvigor210, I: GSE78220), log rank test. **(J)** Differences in TIDE scores of the high and low FS score groups in the TCGA group. **(K)** Correlation analysis between TIDE score and FSscore in the TCGA group. **(L)** The difference in FSscore between drug-resistant and nonresistant patients. ***P < 0.001.

The TCGA database has provided more comprehensive clinical annotations and somatic mutation data for AML patients. We further explored the relationship between ferroptosis-related phenotypes and these clinical characteristics. Through Fisher’s exact test, we analyzed the differences in clinicopathological factors between the high and low FSscore groups (**Figure 7A** and **Table S19**), among which cytogenetic risk, FAB classifications, and white blood cell (WBC) count were significantly different. The high FSscore group had more patients with poor cytogenetic risk, a high WBC count, and M4 and M5 classifications, while the low-risk group had a higher proportion of M1-M3 patients (**Figures 7B–D**). Based on the characteristics of ferroptosis-related phenotypes, we can understand these results well. The immune exclusion and immunosuppression phenotypes corresponding to a high FSscore are accompanied by higher inflammatory cell infiltration, which indicates a higher proportion of WBCs. For example, neutrophils, account for approximately 50-70% of the proportion of WBCs (54), and a high WBC count has been included as a poor prognostic factor for AML (55), so the high FSscore group showed a higher WBC count. Another validation cohort (GSE146173) also had abundant clinical information, and we observed that the high FSscore group showed the same characteristics (**Figures S14A–D** and **Table S20**).

Based on the poor cytogenetic risk of patients in the high FSscore group, we further analyzed the frequency and distribution of somatic mutations between the high and low FSscore groups in the TCGA group using the maftools package. As shown in **Figures 7E, F**, the overall gene mutation frequency of AML patients was not high. The low FSscore group had a lower percentage of mutated samples, and the gene mutation frequency was similar to the average expected value. The high FSscore group had a higher percentage of mutated samples. The mutation rate of the top 20 mutated genes ranged from 2%- 15%, and only a few samples in both groups showed high TMB. Spearman correlation analysis indicated that FSscore and TMB showed a significant negative correlation with each other (**Figure 7G**). Many studies have shown that high TMB in cancer is associated with a better prognosis (56, 57), which may be because tumor cells in patients with high TMB express more immunogens that are recognized by immune T cells and because anti-PD-1/PD-L1 treatment has a better effect in these patients. Therefore, we analyzed the relationship between FSscore and immunotherapy response in the two immune checkpoint inhibitor treatment cohorts (IMvigor210, GSE78220), and we observed that in the two cohorts, patients in the low FSscore group had a better prognosis and that the proportion of patients who responded to immunotherapy was higher (**Figures 7H, I**). This may indirectly prove that patients with a low FSscore have a high TMB and better response to immunotherapy. Moreover, we previously confirmed that the immune exclusion and immunosuppression phenotypes corresponding to a high FSscore are closely related to strong immune escape ability. We again quantitatively analyzed the difference in TIDE score between the high and low FSscore groups. The TIDE score was significantly higher in the high FSscore group (**Figure 7J**). Correlation analysis showed that the FSscore and TIDE score were highly positively correlated (**Figure 7K**). Finally, we explored the relationship between FSscore and chemotherapy resistance. In the GSE146173 cohort, we analyzed the difference in FSscore between drug-resistant and nonresistant patients, and the results showed that the FSscore of drug-resistant patients was significantly higher (**Figure 7L**). A high FSscore is accompanied by the enhanced ability of AML cells to adhere to the bone marrow niche, and it is easy to avoid the attack of chemotherapy drugs, which may be one of the reasons for the occurrence of drug resistance. The above results indicate that the FSscore is closely related to clinical laboratory test indicators, somatic mutations, immunotherapy, immune escape, and drug resistance, which can facilitate clinical treatment decision making.

### Sensitivity analysis of anticancer drugs and prediction of targeted small molecule drugs for ferroptosis-related phenotypes

Patients with a high FSscore may be accompanied by resistance to conventional chemotherapy drugs. Therefore, different clinical treatments are required for patients with different ferroptosis-related phenotypes. In the TCGA and GEO groups, we compared the sensitivity of patients with high and low FSscores to 138 anticancer drugs to assess potentially valuable treatment options. By using the pRRophetic package to predict the IC50 values of different AML patients after drug treatment based on RNA-seq data and performing differential analysis between high and low FSscore groups, we found that the sensitivity of the eight anticancer drugs [A.443654 (pan-AKT inhibitor), ABT.263 (navitoclax, Bcl-2 inhibitor), AG.014699 (rucaparib, PARP inhibitor), AKT inhibitor VIII, AP.24534 (Ponatinib, pan-BCR-ABL inhibitor), AS601245 (JNK inhibitor), AUY922 (luminespib, HSP90 inhibitor) and axitinib (VEGFR inhibitor)] were significantly different in the high and low FSscore groups of the GEO cohort (**Figure S15A**). The sensitivity of six anticancer drugs [ABT.263 (navitoclax, Bcl-2 inhibitor), AG.014699 (rucaparib, PARP inhibitor), AKT inhibitor VIII, AP.24534 (ponatinib, pan-BCR-ABL inhibitor), axitinib (VEGFR inhibitor), and AZ628 (Raf inhibitor) were significantly different in the high and low FSscore groups of the TCGA cohort (**Figure S15B**). Among them, five anticancer drugs (ABT.263, AG.014699, AKT inhibitor VIII, AP.24534, and axitinib) showed significant differences in treatment sensitivity in patients with high and low FSscores in the TCGA and GEO groups, and the IC50 values were higher in patients with high FSscores, indicating that these drugs are more appropriate for the treatment of patients with low FSscore; that is, patients with low FSscores may benefit from treatment with these drugs.

We further performed targeted small molecule drug prediction based on the gene signatures of ferroptosis-related phenotypes, selected highly expressed gene sets in immunosuppression phenotype as upregulated genes, and gene sets expressed at low levels as downregulated genes and uploaded them to the CMap database to analyze potential therapeutic drugs. With the predicted correlation P value < 0.05 as the standard, a total of 44 small molecule drugs and 29 corresponding drug mechanisms were identified (**Figure S15C**). The predicted candidate drugs and potential therapeutic effects can provide references for basic research and clinical trials.

## GPX4 is a potential therapeutic target for AML

**Figure 8:**
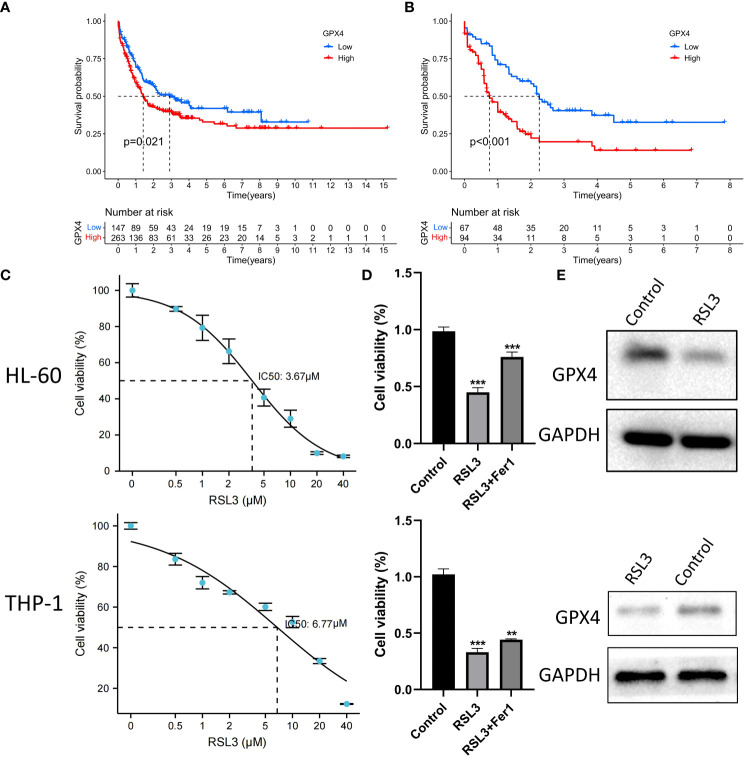
RSL3 inhibits growth of AML cells. **(A, B)** Differences in the survival of patients in high and low expression of GPX4 in the GEO and TCGA groups, A: GEO group, B: TCGA group, log rank test. **(C)** HL-60 cells and THP-1 cells were treated with RSL3 at the indicated doses for 48 h and cell viability was assayed using a CCK-8 kit. **(D)** HL-60 cells and THP-1 cell were treated with RSL3(HL-60: 5 μM; THP-1: 10μM) with or without ferrostatin-1 for 48 h and cell viability was assayed, ***P < 0.001. **(E)** Protein level of *GPX4* in HL-60 cells and THP-1 cells treated with RSL3 (HL-60: 5 μM; THP-1: 10μM) or control for 48 hours. **P < 0.01.

We observed that *GPP4* is highly expressed in AML patients and has the highest expression level in immunosuppressive phenotype, suggesting that *GPX4* may be a potential therapeutic target. Both in GEO group and TCGA group, the prognosis of patients with high expression of *GPX4* was significantly worse (**Figures 8A, B**). We further used RSL3, a targeted inhibitor of *GPX4*, in AML cell line HL-60 to explore the biological effects of inducing ferroptosis. Cell viability assay showed that RSL3 inhibited HL-60 cells and THP-1 cells in a dose-dependent manner (**Figure 8C**), and the addition of iron death inhibitor (ferrostatin-1) could partially save cell viability (**Figure 8D**). Western blot analysis showed that RSL3 could significantly reduce the expression of *GPX4* (**Figure 8E**), indicating that inhibiting *GPX4* could promote the death of HL-60 cells and THP-1 cells by inducing ferroptosis.

## Discussion

AML, a hematological malignant tumor, has a high degree of heterogeneity. The complex and dynamic clonal architecture is the main reason for the refractoriness of AML (58–60). According to the FAB classification, patients can be divided into eight classifications (M0-M7) (61). AML is highly malignant, with a five-year survival rate of less than 30%, and is the most common leukemia among the elderly (62). Chemotherapy and hematopoietic stem cell transplantation (HSCT) are the most common treatments for AML (63), but drug resistance and recurrence lead to unsatisfactory treatment outcomes (64). Therefore, the development of new treatment methods to improve the effects of AML treatment has important clinical significance. As a new type of cell death mode, ferroptosis has a profound impact on the development and treatment of many diseases, especially cancers (65). Ferroptosis is closely related to the biological characteristics of the TME. Hypoxia induces the production of ROS, and the activation of lipid metabolism and the immune response create conditions for ferroptosis (66). These factors all show that tumor cells are prone to ferroptosis. Therefore, strategies for inducing ferroptosis in tumor cells and weakening the protective mechanism should have the most direct clinical value for cancer treatment, with the ultimate goal of promoting tumor cell death. In this study, we explored the genomic characteristics of FRGs in AML and their correlation with the TME and prognosis of AML patients and found that most FRGs showed high expression of mRNA levels and interactions at the protein level and were also were significantly related to the prognosis of patients. FRGs participate in many metabolic-related signaling pathways, and high expression of these FRGs may be one of the factors promoting the growth of AML tumor cells and the deterioration of the TME.

FRGs may also collaborate to induce more potent effects on AML. We observed three ferroptosis-related phenotypes in the ferroptosis-related molecular patterns and genomic subtypes; they were defined as the immune activation phenotype, immune exclusion phenotype, and immunosuppression phenotype. These phenotypes showed significant differences in characteristics such as immunity, inflammation, lipid metabolism, and prognosis and represented a progressively deteriorating pathological state. The occurrence of ferroptosis is closely related to biological factors such as immunity, inflammation, and metabolism. For example, the activation of immune cells, such as CD8+ T cells, through the secretion of INF-γ downregulates the expression of components of system XC^−^, such as *SLC3A2* and *SLC7A11*, to inhibit the uptake of cystine by tumor cells, thereby promoting ferroptosis induced by the depletion of glutathione (67). Ferroptotic cancer cells containing immunogens can be recognized and engulfed by macrophages (21). Ferroptosis and inflammation are also complementary. The occurrence of ferroptosis increases the expression of *PTGS2* encoding *COX2* and further promotes the metabolism of AA to increase the secretion of inflammatory signal molecules (14); treatment of cells with the inflammatory cytokine TNF leads to continuous downregulation of *GPX4* to induce ferroptosis (68). Unsaturated fatty acids act as peroxidation substrates for ferroptosis and are also important regulators of inflammatory processes along with their metabolic enzymes (69). Samples with different ferroptosis-related phenotypes also showed different sensitivities to ferroptosis. For the immune activation phenotype, a large number of immune cells can promote the ferroptosis of tumor cells through different induction methods; for the immunosuppression phenotype, the hypoxia of the TME increases the generation of ROS, and the high development of inflammation can also increase the sensitivity of tumor cells to ferroptosis; the immune exclusion phenotype is accompanied by immune escape and decreased inflammation, and the sensitivity to ferroptosis is relatively low. Moreover, the enrichment of unsaturated fatty acids in immune activation and immunosuppression phenotypes provides conditions for the occurrence of ferroptosis. Therefore, these results suggest that different methods can be used to induce ferroptosis in patients with different ferroptosis-related phenotypes. For patients with immune activation phenotype, we can induce ferroptosis in leukemia cells by stimulating immune cells such as CD8+ T cells to secrete more INF-γ, and inhibit the expression of ferroptosis inhibitors such as *GPX4* and *DHODH*. For immunosuppression phenotype, we can induce ferroptosis by targeting enhanced lipid peroxidation. For patients with immune exclusion phenotype, ferroptosis can be induced in leukemia cells by simultaneously activating the immune system and enhancing lipid peroxidation at the same time. These insights may provide new ideas for the treatment of drug resistance caused by clinical chemotherapy and targeted therapy.

In the subsequent analysis, we further analyzed the immune escape levels of the three ferroptosis phenotypes. Unlike solid tumors that can block the infiltration of immune cells through stromal cells to produce immune exclusion (70), AML, as a hematological tumor, can avoid the attack of immune cells through adhesion to the niche (71). The expression levels of immune checkpoints in patients with immune activation and immune suppression phenotypes are significantly higher, and the signaling pathways related to immune escape are significantly activated; they also show consistency in the transcriptome. In terms of the mechanism of promoting immune escape, tumor cells with an immune exclusion phenotype mainly prevent the infiltration of a large number of immune cells by increasing adhesion and promoting the release of inflammatory signals. On the other hand, tumor cells of the immune exclusion phenotype mainly exploit the worsening chronic inflammatory microenvironment to create favorable living conditions for themselves (72), and the immune cells of this phenotype are in a suppressed state. These results suggest that for patients with different ferroptosis-related phenotypes, AML patients can be treated by drugs that induce ferroptosis or suppress immune escape according to the corresponding phenotypic characteristics. To judge the individual characteristics of a single patient more accurately, we constructed the FSscore to quantify the degree of development of ferroptosis-related phenotypes. The FSscore can quantitatively evaluate the pathological status of patients and also has good prognostic value. We confirmed this in two groups and two other cohorts that used different sequencing methods and showed certain prognostic prediction accuracy across cancers. In terms of various pathological characteristics, a high FSscore is highly positively correlated with inflammation development, immune escape, lipid metabolism, anti-immunotherapy, chemotherapy resistance, etc., and negatively correlated with tumor mutation burden. These results all show the reference value of the FSscore for the evaluation of individual characteristics and clinical treatment of AML patients.

Finally, to explore the clinical treatment options for patients with different ferroptosis-related phenotypes, we used drug prediction to identify five anticancer drugs and nine small molecule drugs that may have therapeutic benefits for patients with low FSscores and 20 small molecule drugs that may have therapeutic benefits for patients with high FSscores. Among them, ABT.263 (navitoclax) has been confirmed to be effective for the treatment of AML in cell and mouse experiments (73–76). The combination of hyperforin and Akt inhibitor AKT inhibitor VIII significantly promotes the apoptosis of AML U937 cells (77). Second-generation tyrosine kinase inhibitors (TKIs), such as ponatinib (AP.24534), are also widely used in hematological malignancies (78). Axitinib effectively inhibits BCR-ABL1 (T315I) to treat chronic myeloid leukemia (79). Research on these drugs suggests that they can improve the treatment of hematological tumors and provide a reference for further basic research and clinical trials. In addition, we used GPX4 targeting inhibitor RSL3 to reduce the protein level of GPX4 and promote AML cell death by inducing ferroptosis, indicating that GPX4 is a potential target for AML treatment.

In summary, we found three ferroptosis-related phenotypes in AML patients based on FRG analysis and revealed the TME characteristics (such as immune escape, inflammation development, and lipid metabolism) of samples with different phenotypes. The FSscore can improve the assessment of the pathological state and prognosis of AML patients and provides reference value for the establishment of more personalized clinical treatment plans. Moreover, compared with other studies on ferroptosis in AML, our project also has its own advantages and limitations. For example, compared with the data analysis of Zhou et al. (80), our study not only used more AML samples and multi-omics data such as transcriptome, copy number variation, and gene mutation, but also verified the existence of three ferroptosis-related phenotypes in both microarray and high-throughput sequencing types, and explored the differences in TME and clinical pathological characteristics among them, the constructed scoring system can also accurately predict the prognosis of AML patients and provide some insights for the therapeutic evaluation. However, compared with the research of Yusuf et al. (81), our exploration of ferroptosis mainly focuses on the analysis of big data of bioinformatics, but there are obvious deficiencies in the exploration of regulation mechanism, which is the direction of our follow-up study, linking more regulatory mechanisms of ferroptosis in AML cells with bioinformatics data.

## Conclusions

This project revealed that the occurrence of ferroptosis is closely related to the complex pathological changes in the TME. Patients with different TME features show differences in terms of ferroptosis sensitivity. Immune cell infiltration, inflammation development and lipid metabolism are important regulatory factors that affect the occurrence of ferroptosis. The comprehensive analysis of ferroptosis-related molecular patterns in individual patients can provide references for the clinical evaluation of patient pathological characteristics and the design of personalized treatment plans.

## Data availability statement

The original contributions presented in the study are included in the article/**Supplementary Material**. Further inquiries can be directed to the corresponding authors.

## Author contributions

F-MZ research design and drafing the manuscript. F-YY helping to revision the manuscript. H-BZ, JZ, JLiu, J-YJ, SX, WW, X-RZ, X-XC, Y-LY, YC, JLin, S-QL, NZ and M-YL assisted bioinformatic and statistical analysis. BH revision of the manuscript and writing guidance. X-ZW review and revision of the manuscript and writing guidance. All authors contributed to the article and approved the submitted version.

## Funding

The study was funded by the National Natural Science Foundation of China (81860034, 82160405, 82160038).

## Conflict of interest

The authors declare that the research was conducted in the absence of any commercial or financial relationships that could be construed as a potential conflict of interest.

## Publisher’s note

All claims expressed in this article are solely those of the authors and do not necessarily represent those of their affiliated organizations, or those of the publisher, the editors and the reviewers. Any product that may be evaluated in this article, or claim that may be made by its manufacturer, is not guaranteed or endorsed by the publisher.
