# Supplementary material for: Ferroptosis-related molecular patterns reveal immune escape, inflammatory development and lipid metabolism characteristics of the tumor microenvironment in acute myeloid leukemia
Source: Front Oncol. 2022 Nov 28;12:888570. doi: 10.3389/fonc.2022.888570 (PMC9742468; doi:10.3389/fonc.2022.888570)
Supplement: Supplementary file 1 [file DataSheet_1.pdf]

Figure S1. The workflow of this project.

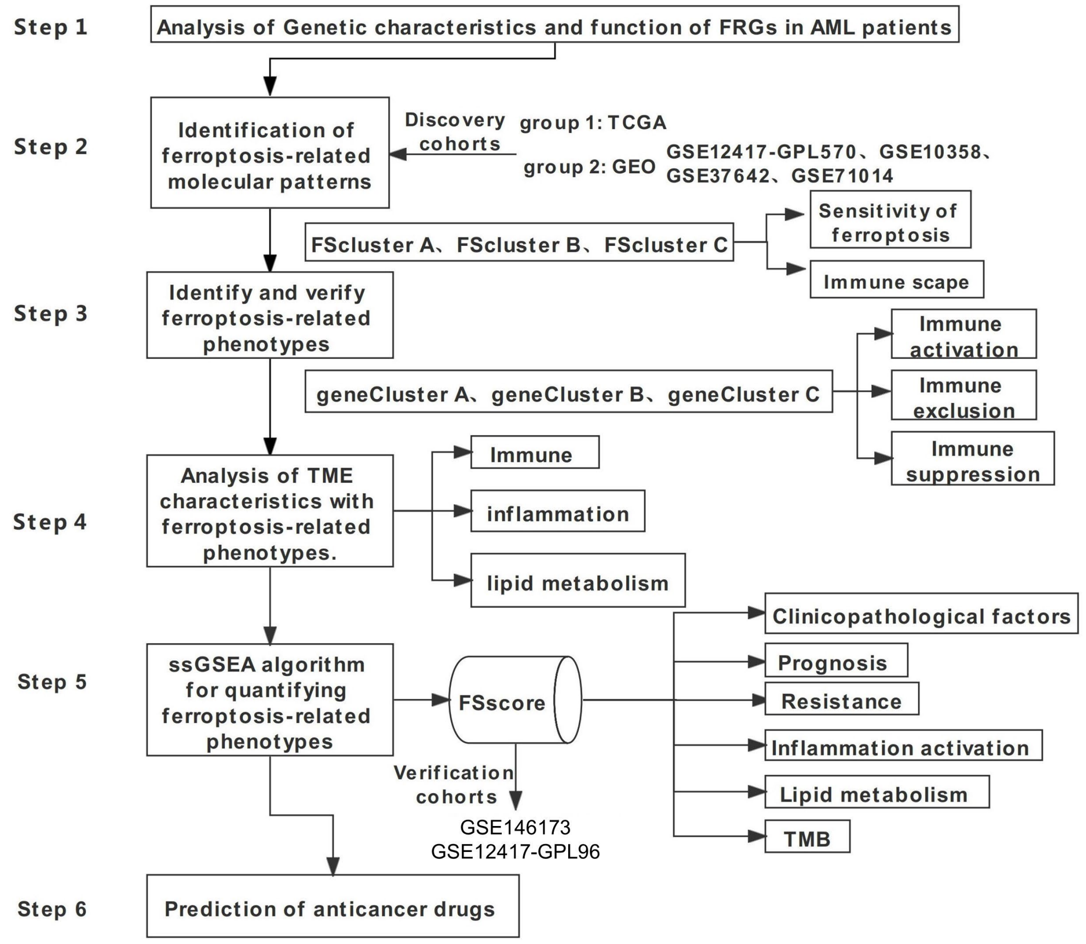

Figure S2. Identification of ferroptosis-related molecular patterns.

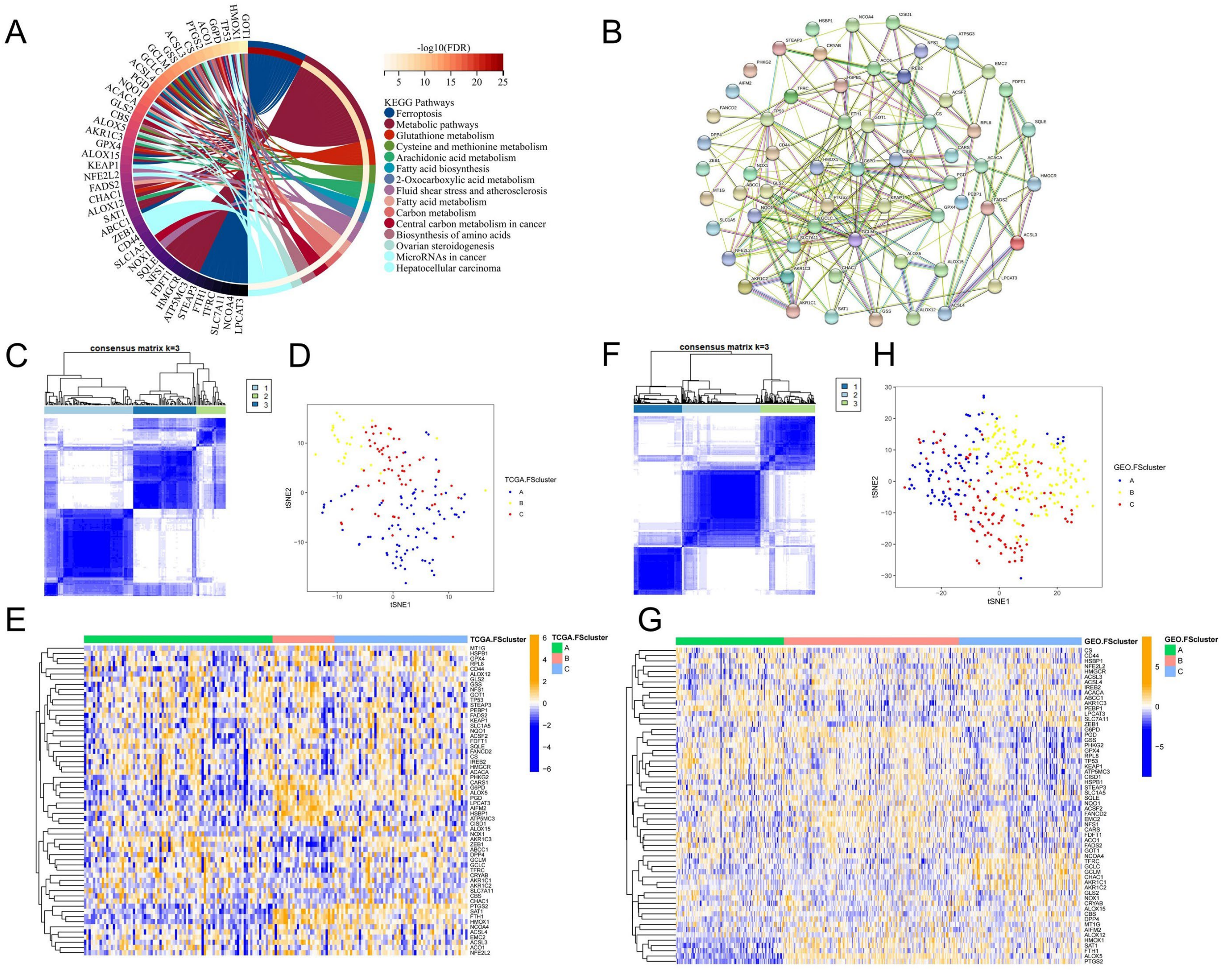

(A) KEGG pathway enrichment analysis showed the enriched pathways and related genes, FDR < 0.05. (B) PPI network of FRGs. (C) Consensus matrices of the TCGA group for k = 3. (D) t-SNE verified the reliability of unsupervised clustering in the TCGA group. (E) The expression of FRs in different ferroptosis-related molecular pattern groups in the TCGA group. (F) Consensus matrices of the GEO group for k =3. (G) The expression of FRs in different ferroptosis-related molecular pattern groups in the GEO group. (H) t-SNE verified the reliability of unsupervised clustering in the GEO group.

Figure S3. Biological characteristics analysis of ferroptosis-related molecular patterns in the TCGA group.

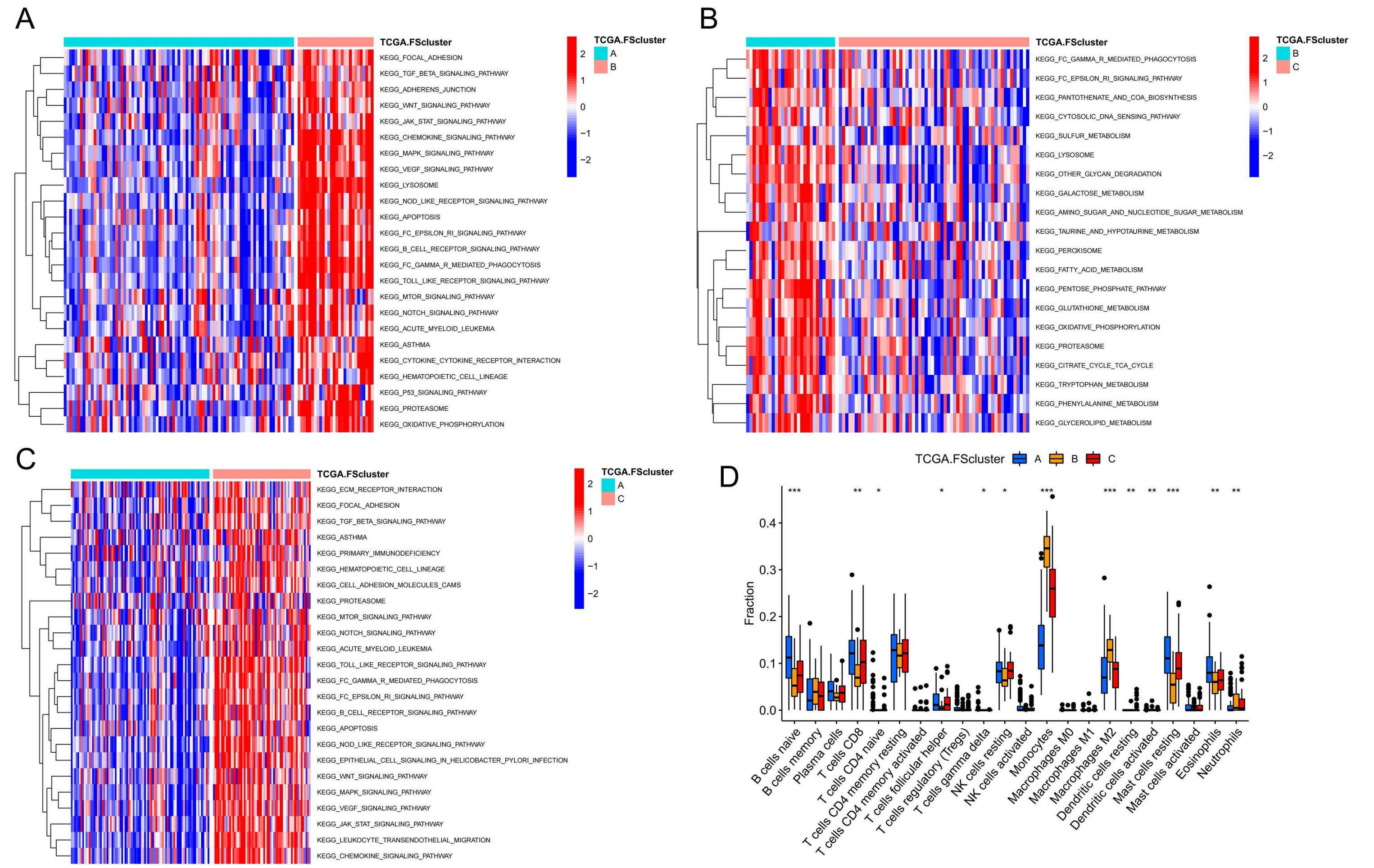

(A-C) GSVA showed the activation levels of biological pathways in different ferroptosis-related molecular pattern groups. (D) The infiltration ratio of various TME cells in three ferroptosis-related molecular pattern groups, Kruskal–Wallis test, * P < 0.05; ** P < 0.01; * P < 0.001.

Figure S4. Lipid metabolism characteristics of ferroptosis-related molecular patterns in the GEO group.

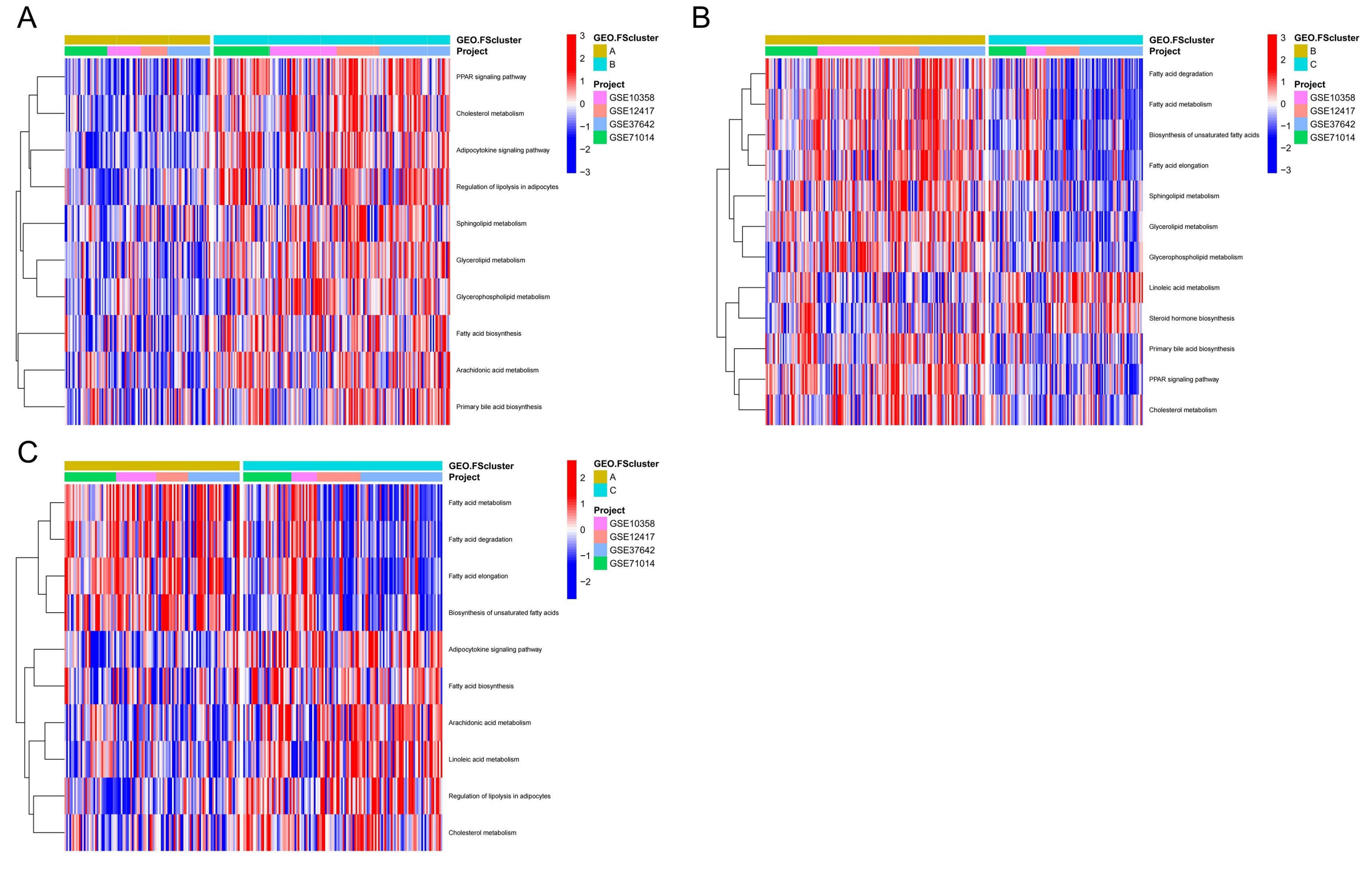

(A-C) GSVA showed the activation levels of lipid metabolism pathways in different ferroptosis-related molecular pattern groups.

Figure S5. Lipid metabolism characteristics of ferroptosis-related molecular patterns in the TCGA group.

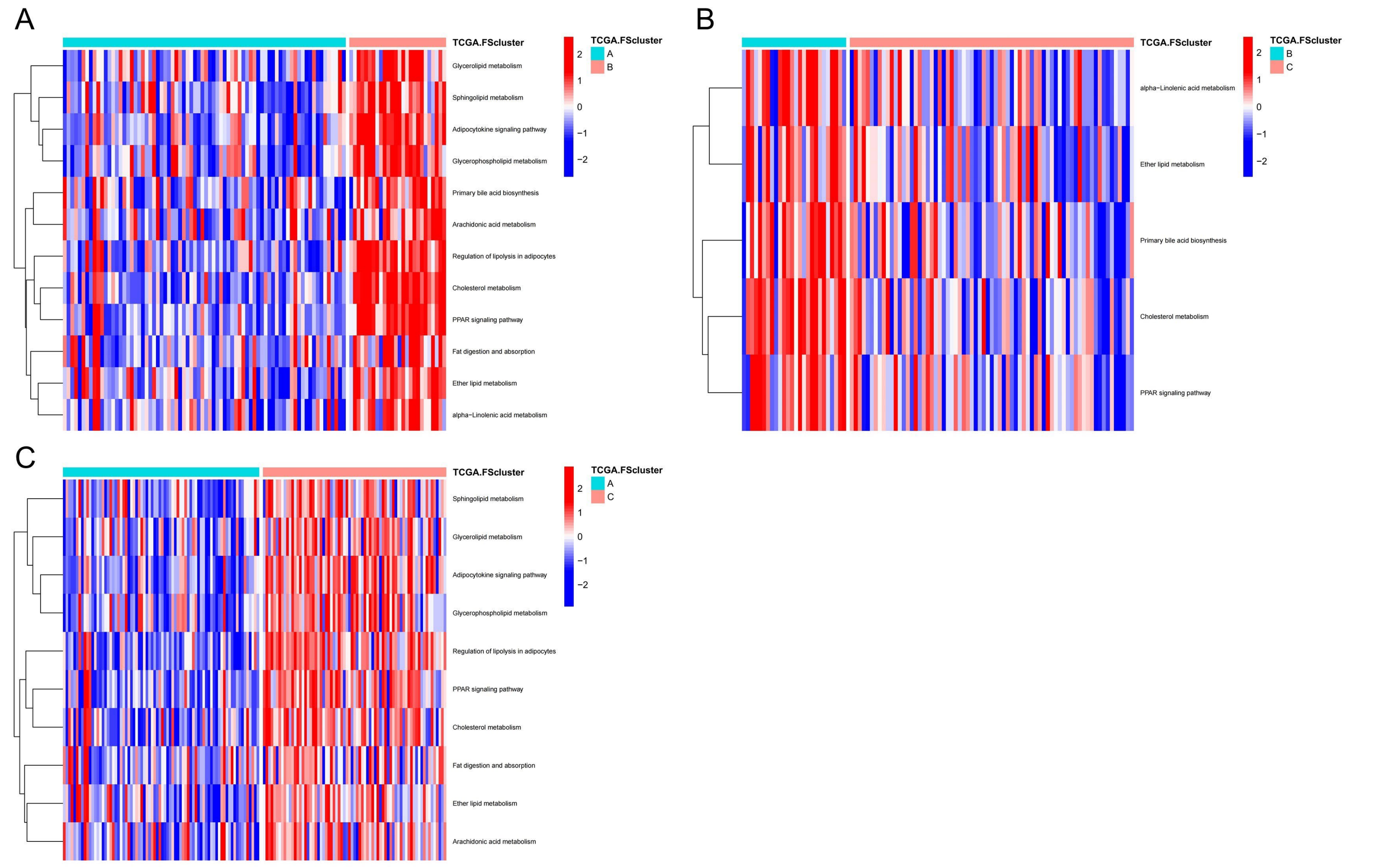

(A-C) GSVA showed the activation levels of lipid metabolism pathways in different ferroptosis-related molecular pattern groups.

Figure S6. Prognostic analysis of BUFAscore and identify the subtypes of FScluster B in GEO group.

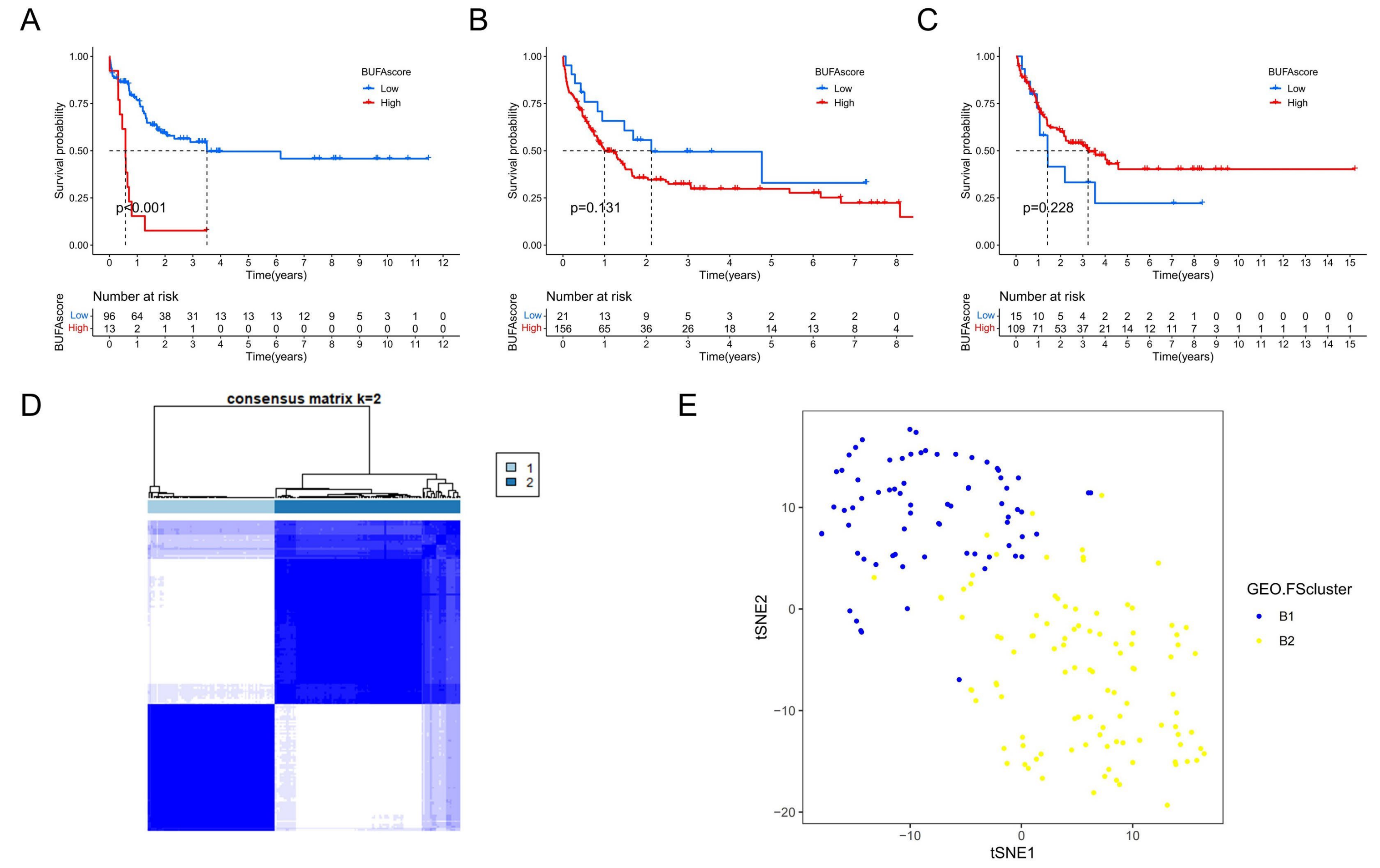

(A-C) The relationship between BUFAscore and prognosis in each ferroptosis-related molecular pattern in the GEO group, log rank test, A: FScluster A; B: FScluster B; C: FScluster C. (D) Consensus matrices of the FScluster B in GEO group for k = 3. (E) t-SNE verified the reliability of unsupervised clustering

Figure S7. Analysis of other factors affecting ferroptosis in the TCGA group.

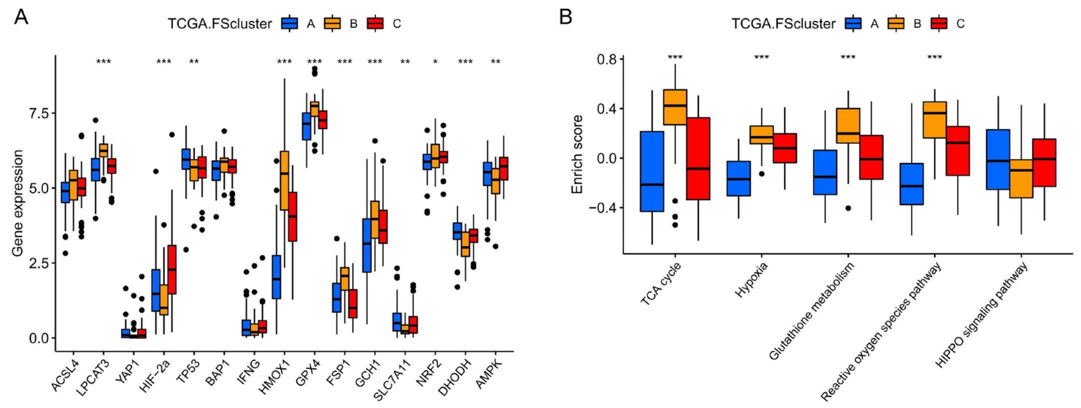

(A-B) Differences in genes and signaling pathways that affect ferroptosis among different ferroptosis-related molecular pattern groups, Kruskal–Wallis test, * P < 0.05; ** P < 0.01; * P < 0.001.

Figure S8. Identification of genomic subtypes.

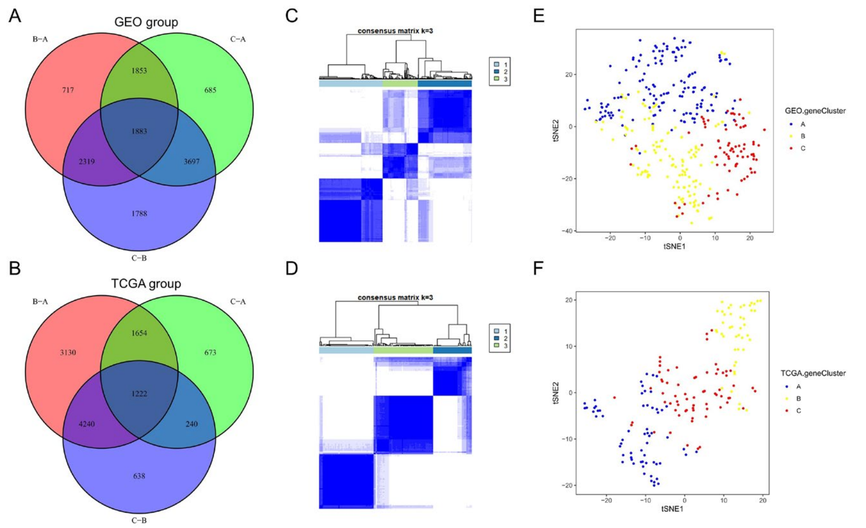

(A-B) Differentially expressed genes shared between the molecular patterns in the GEO and TCGA groups. (C) Consensus matrices of the GEO group for k = 3. (D) Consensus matrices of the TCGA group for k = 3. (E-F) t-SNE verified the reliability of unsupervised clustering in the GEO and TCGA groups.

Figure S9. Distribution characteristics and survival analysis of genomic subtypes.

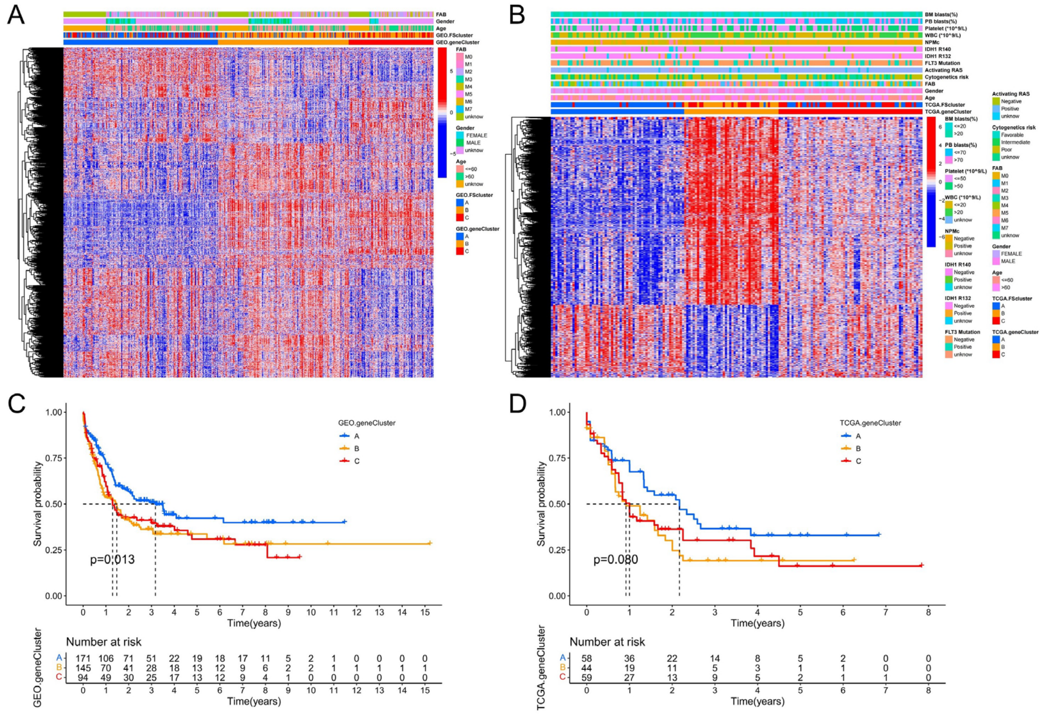

(A-B) Differences in patient distribution, clinicopathological factors and shared differential gene expression of different genomic subtypes in the GEO and TCGA groups. (C-D) Differences in the survival of patients with different genomic subtypes in the GEO and TCGA groups, log rank test.

Figure S10. Biological characteristics analysis of genomic subtypes in the GEO group.

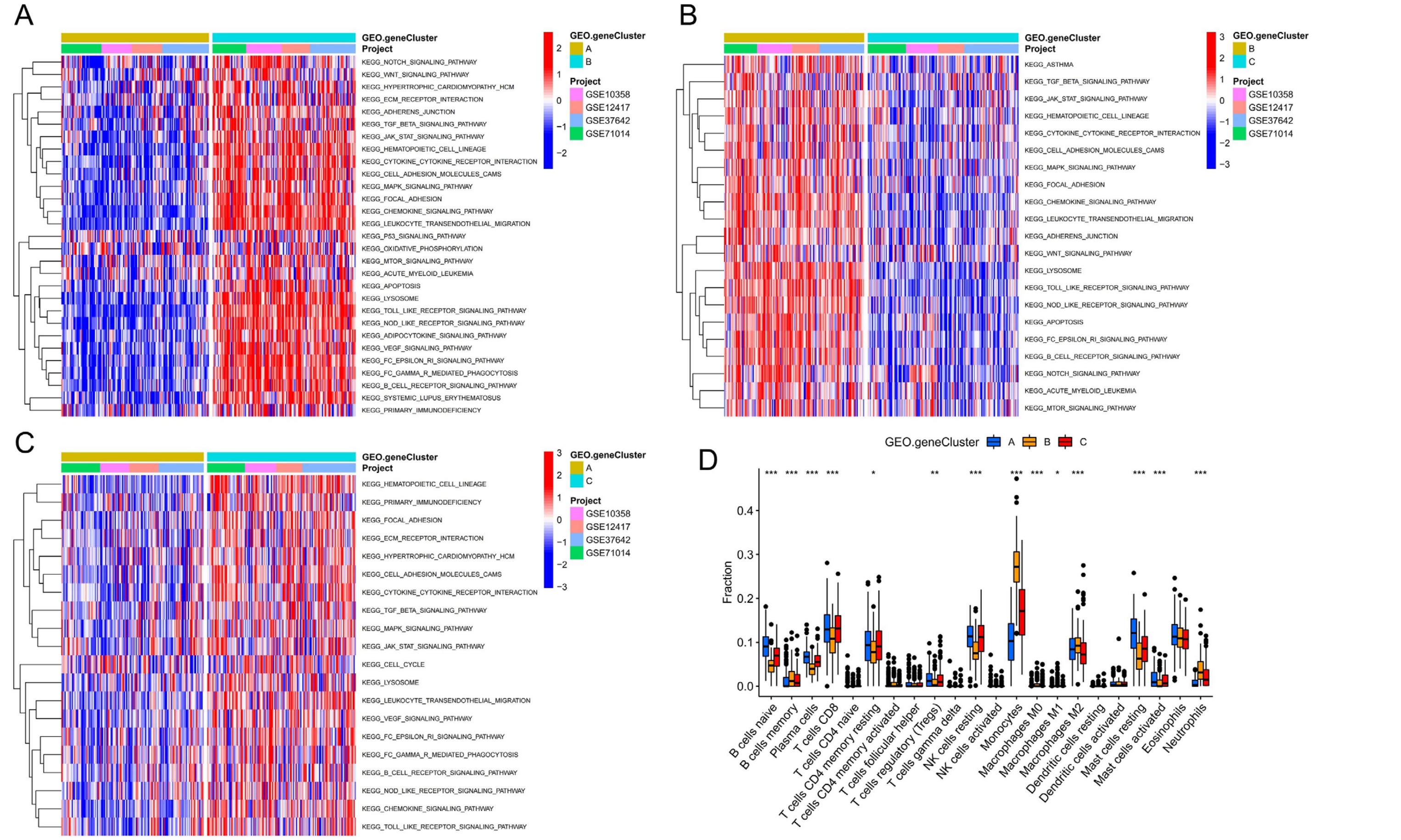

(A-C) GSVA showed the activation levels of biological pathways in different genomic subtype groups. (D) The infiltration ratio of various TME cells in three genomic subtypes, Kruskal–Wallis test, * P < 0.05; ** P < 0.01; * P < 0.001.

Figure S11. Biological characteristics analysis of genomic subtypes in the TCGA group.

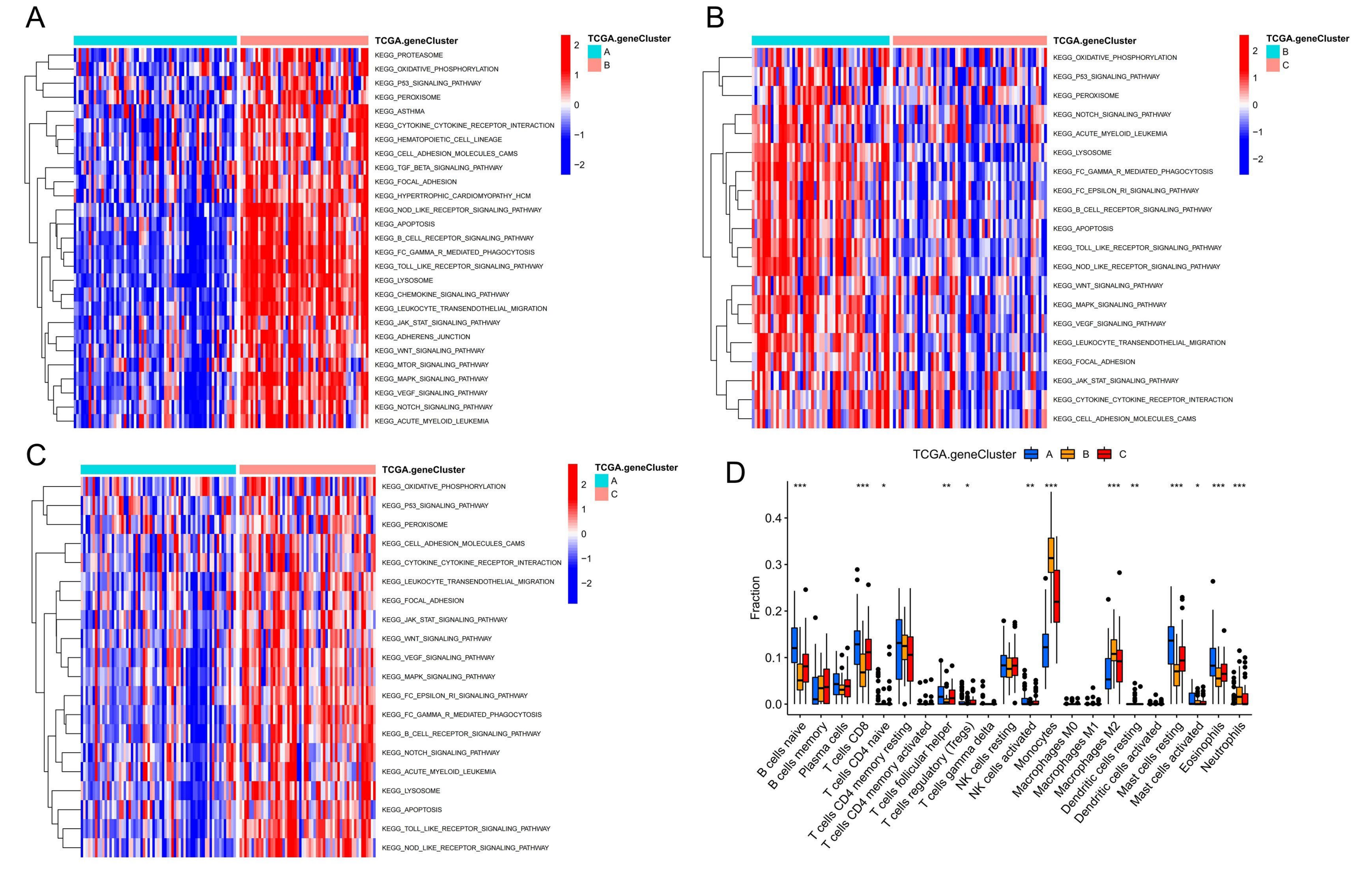

(A-C) GSVA showed the activation levels of biological pathways in different genomic subtypes. (D) The infiltration ratio of various TME cells in three genomic subtypes, Kruskal–Wallis test, * P < 0.05; ** P < 0.01; * P < 0.001.

Figure S12. Characteristic analysis of the TME with ferroptosis-related phenotypes in the TCGA group.

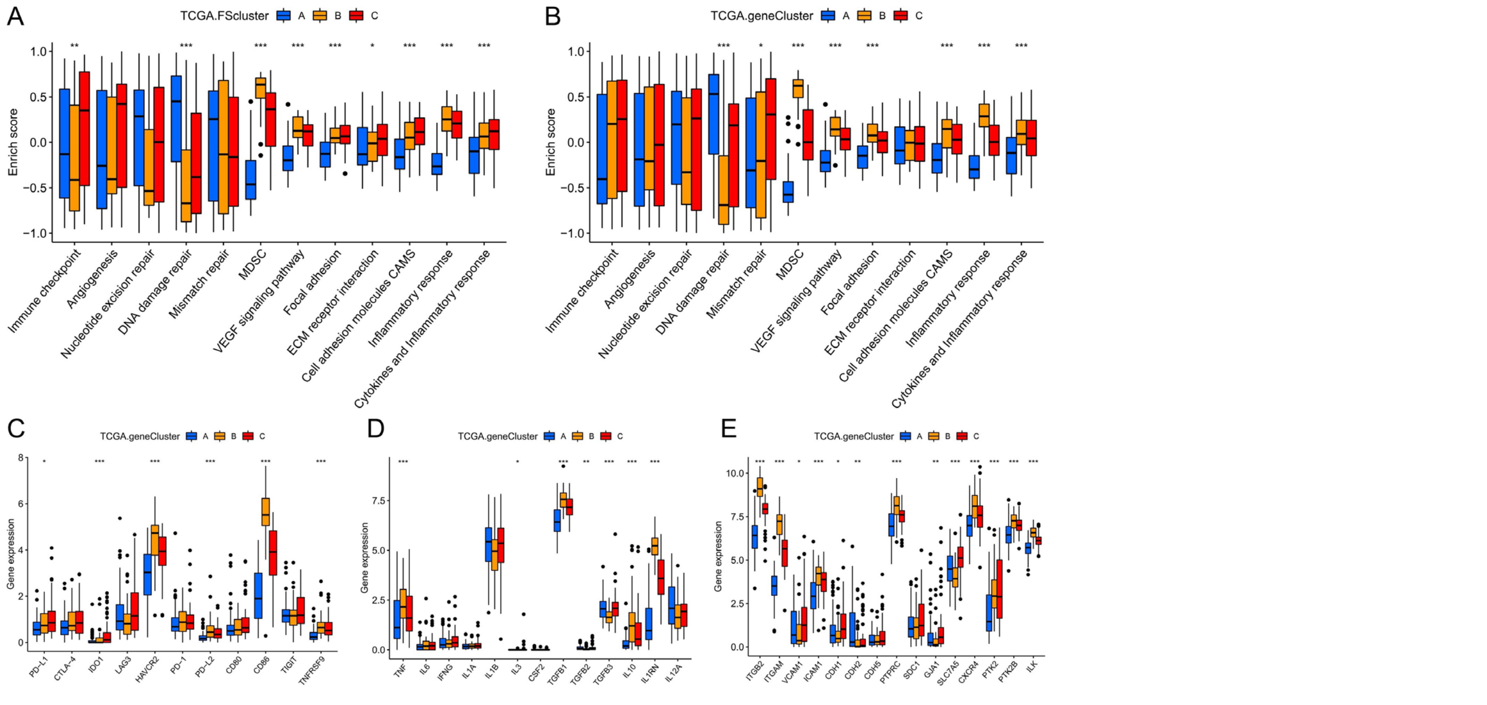

(A-B) Differences in pathway signatures related to tumor development and the infiltration level of MDSCs in the TME of ferroptosis-related molecular pattern groups and genomic subtype groups in the TCGA group. (C-E) Differences in gene signatures related to tumor development in the TME of ferroptosis-related molecular patterns and genomic subtypes in the TCGA group. C: immune checkpoint-related genes, D: cytokines related to inflammation associated with AML, E: cell adhesion molecules associated with AML. Kruskal–Wallis test, * P < 0.05; ** P < 0.01; * P < 0.001.

Figure S13. Prognostic value of the FSscore in all independent cohorts of the GEO group and correlation analysis between characteristics of TME and FSscore in the TCGA group.

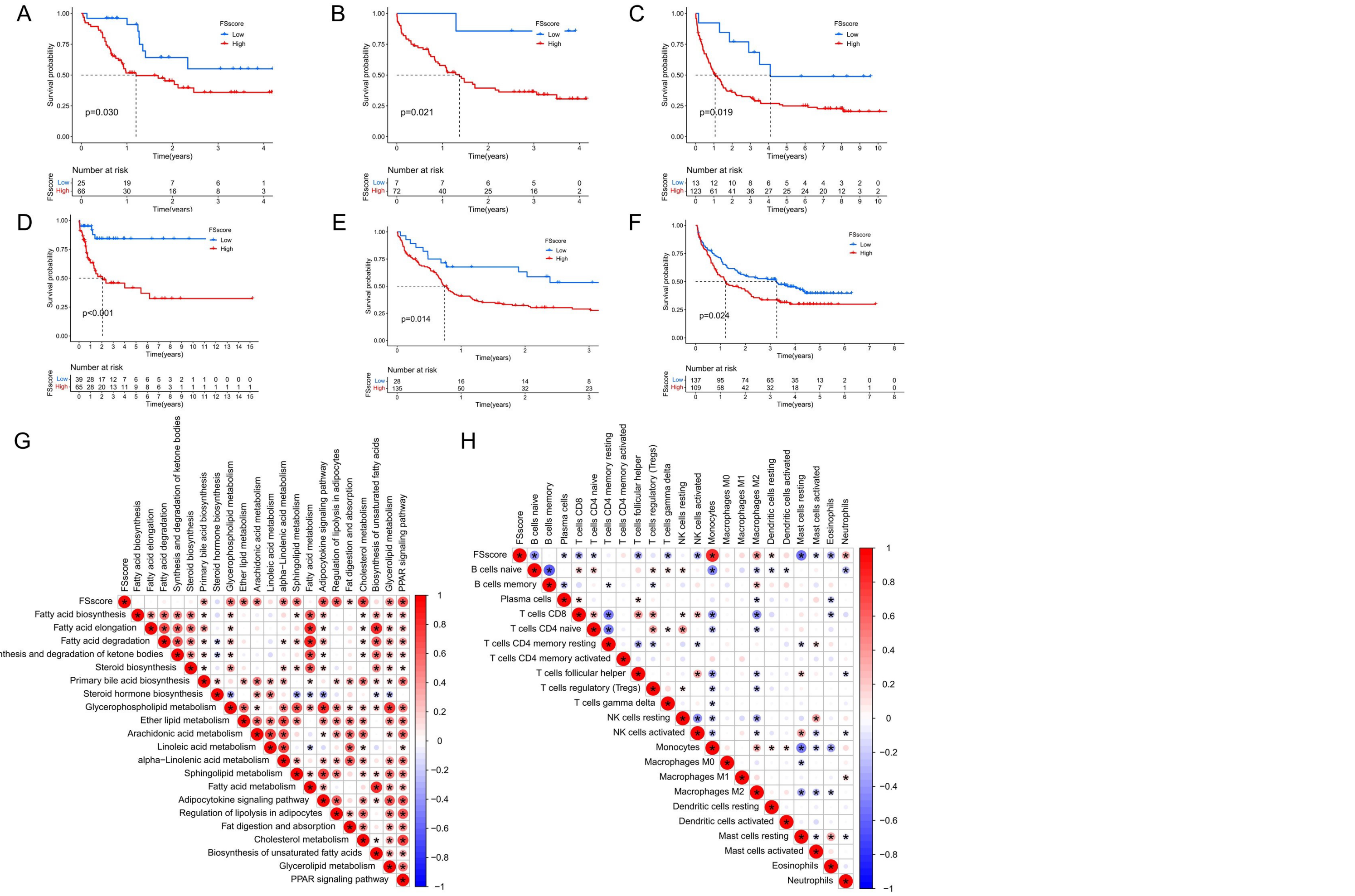

(A-D) Prognostic value of the FSscore in all independent cohorts of the GEO group, A: GSE130358, B: GSE12417-GPL570, C: GSE37642, A: GSE71014, log rank test. (E-F) Differences in the survival of patients in the high and low FSscore groups of the validation cohorts, E: GSE12417-GPL96, F: GSE146173, log rank test. (G) Correlation analysis between the levels of TME cell infiltration and FSscore in the TCGA group. (H) Correlation analysis between levels of lipid metabolism and FSscore in the TCGA grou

Figure S14. Validation of the relationship between clinicopathological factors and FSscore.

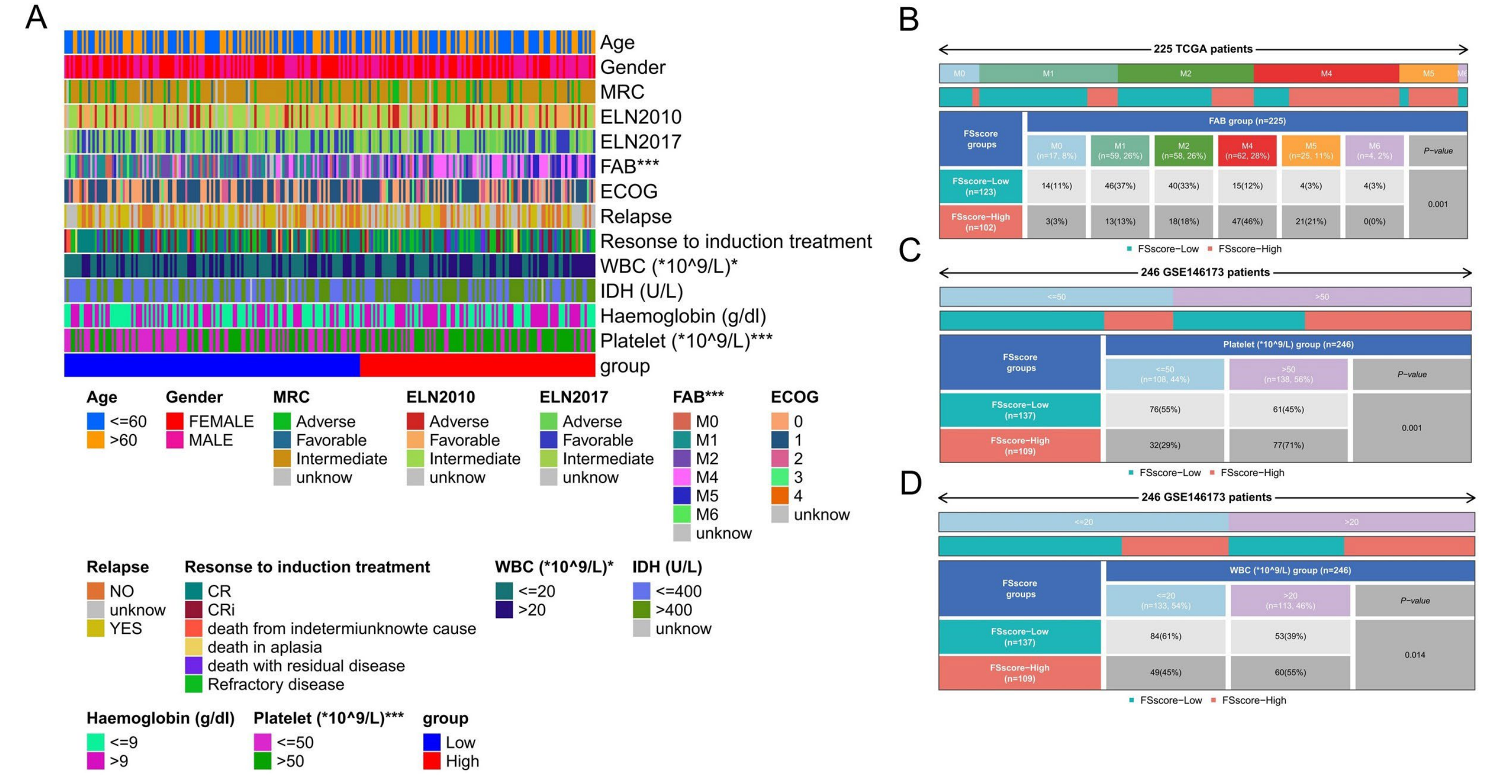

(A-D) Correlation analysis between clinicopathological factors and FSscore in the GSE146173 cohort, Fisher's exact test, * P < 0.05; ** P < 0.01; * P < 0.001, WBC: white blood cell, MRC: myelodysplasia-related changes, ELN: European Leukemia Network, ECOG: Eastern Cooperative Oncology Group, IDH: isocitrate dehydrogenase.

Figure S15. Prediction of drugs for treating patients with different ferroptosis-related phenotypes.

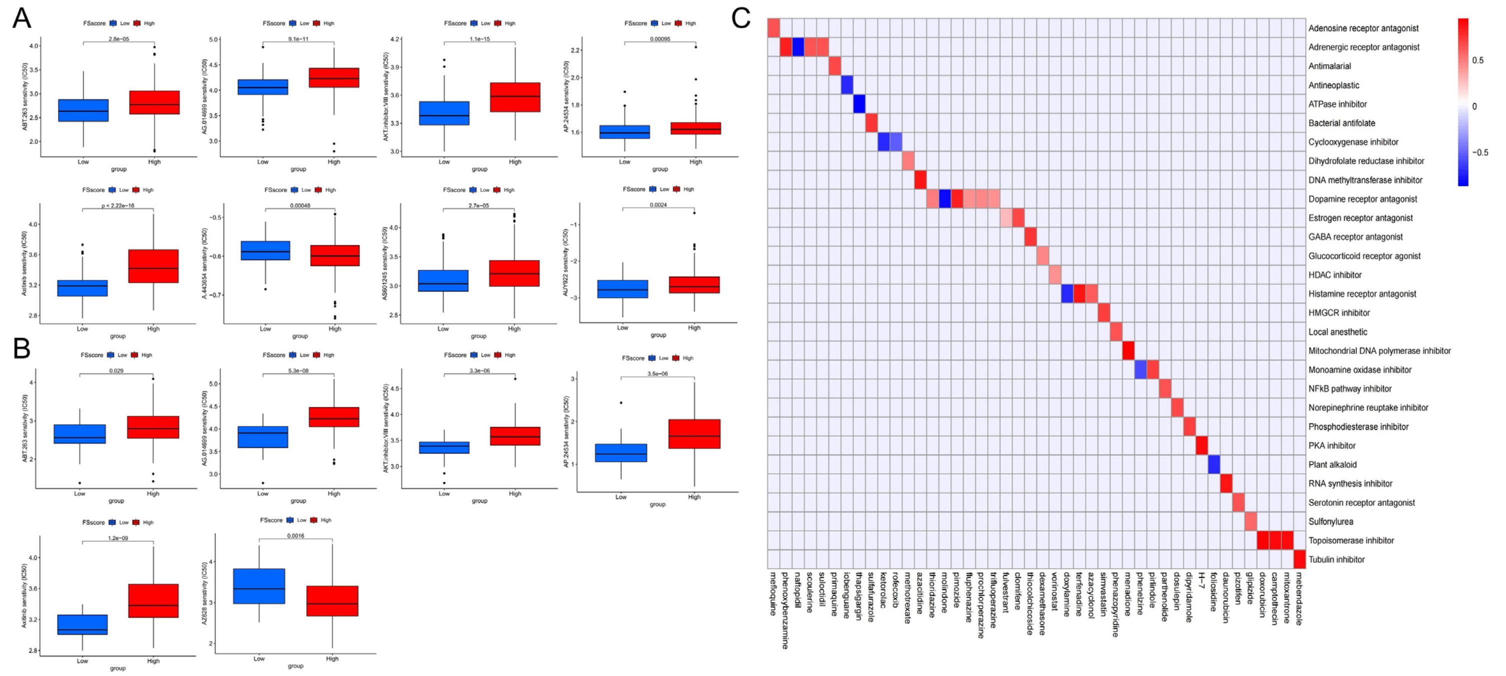

(A-B) Sensitivity analysis of anticancer drugs in the high and low FSscore groups. A: GEO group, B: TCGA group, Wilcoxon test. (C) Heatmap showing the small molecule drugs and corresponding drug mechanisms. Red represents the phenotype associated with a high FSscore, and blue represents the phenotype associated with a low FSscore; the darker the color is, the higher the correlation.
